# Trophoblast cell-surface antigen 2 expression in digestive neoplasms: a promising target for antibody-drug conjugates

**DOI:** 10.1093/oncolo/oyaf320

**Published:** 2025-10-16

**Authors:** Jinru Yang, Fangyuan Zhang, Xing Cai, Zixuan Ding, Conghua Xie, Hong Qiu

**Affiliations:** Department of Oncology, Hubei Key Laboratory of Tumor Biological Behaviors, Hubei Cancer Clinical Study Center, Zhongnan Hospital of Wuhan University, Wuhan, Hubei 430071, P.R. China; Department of Oncology, Tongji Hospital Affiliated to Tongji Medical College of Huazhong University of Science and Technology, Wuhan, Hubei 430030, P.R. China; Cancer Center, Union Hospital, Tongji Medical College, Huazhong University of Science and Technology, Wuhan, Hubei 430022, P.R. China; The First School of Clinical Medicine of Lanzhou University, Lanzhou, Hubei 730000, P.R. China; Department of Oncology, Hubei Key Laboratory of Tumor Biological Behaviors, Hubei Cancer Clinical Study Center, Zhongnan Hospital of Wuhan University, Wuhan, Hubei 430071, P.R. China; Wuhan Research Center for Infectious Diseases and Cancer, Chinese Academy of Medical Sciences, Wuhan, Hubei 420071, P.R. China; Department of Oncology, Tongji Hospital Affiliated to Tongji Medical College of Huazhong University of Science and Technology, Wuhan, Hubei 430030, P.R. China

**Keywords:** trophoblast cell-surface antigen 2, digestive tumor, targeted therapy, antibody-drug conjugate

## Abstract

**Background:**

Trophoblast cell-surface antigen 2 (Trop2), a transmembrane glycoprotein overexpressed in multiple cancers, plays crucial roles in tumor progression and therapy resistance, yet its expression patterns and clinical significance in digestive cancers remain incompletely characterized.

**Methods:**

This retrospective study analyzed a consecutive cohort of 2370 patients with histologically confirmed digestive cancers (804 gastric [GC], 1,384 colorectal [CRC], and 182 pancreatic cancers [PC]). Comprehensive clinicopathological data were systematically collected. Trop2 expression was quantitatively evaluated by immunohistochemistry and classified into Trop2-negative, Trop2-low, and Trop2-high based on the product of staining intensity and the proportion of positive tumor cells. Statistical analyses included univariate and multivariate logistic regression were used to identify significant clinicopathological and molecular predictors of Trop2 expression patterns. Univariate and multivariate logistic regression analyses were used to explore the relationship between Trop2 expression status (positive [Trop2 intensity ≥ 2] vs. negative) and various clinicopathological features of different tumor types.

**Results:**

Trop2 was widely expressed in digestive cancers, with highest prevalence in PC and GC. Multivariate analysis revealed distinct Trop2 expression patterns in gastrointestinal malignancies. At the pan-cancer level, Trop2 expression significantly correlated with tumor type, (signet ring cell carcinoma) SRCC, vascular invasion (VI) and perineural invasion. Notably, GC showed independent associations with SRCC and intestinal-type Lauren classification; CRC showed VI as the predominant factor associated with Trop2 expression; while PC demonstrated unique correlations with female sex and T1 stage. These findings highlight tumor-type specific regulation of Trop2, providing critical insights for prognostic assessment and targeted therapy.

**Conclusion:**

Trop2 is a promising biomarker for tumor aggressiveness and a potential target for antibody-drug conjugates (ADCs) in digestive cancers, particularly in SRCC-rich, metastatic, and invasive subtypes. These findings provide strong rationale for stratifying patient populations in future clinical investigations of Trop2-directed ADC therapies.

Implications for Practice This study underscores the potential of trophoblast cell-surface antigen 2 (Trop2) as a significant biomarker for tumor aggressiveness in digestive cancers. Its expression, notably in pancreatic cancers and gastric cancers, correlates with key clinicopathological features such as tumor type, stage, and invasion patterns. The distinct Trop2 expression profiles observed in gastric, colorectal, and pancreatic cancers suggest the need for tailored therapeutic strategies. Trop2-targeted therapies, particularly antibody-drug conjugates , may offer a promising approach for treating aggressive subtypes, including signet ring cell carcinoma-rich and metastatic tumors. These findings emphasize the importance of incorporating Trop2 expression as a prognostic tool and therapeutic target in clinical settings.

## Introduction

Digestive system cancers are among the most common malignancies worldwide, with increasing incidence rates.^1^ Treatment strategies depend on tumor type, stage, molecular characteristics, and patient status.^2^ Early diagnosis and surgery are crucial, while systemic therapy remains the mainstay for advanced cases. Although targeted and immunotherapies have improved outcomes in some patients,^3^ digestive cancers, particularly pancreatic cancer (PC), still pose significant therapeutic challenges. In recent years, antibody-drug conjugates (ADCs) have emerged as a promising treatment option.^4^^,^^5^ Antibody-drug conjugates are a type of precision anti-cancer drug that couples targeted monoclonal antibodies with highly effective cytotoxic drugs through chemical linkers. It combines the targeting properties of antibodies with the strong killing power of chemotherapy drugs. ADC selectively kills tumor cells and reduces systemic toxicity by partially binding to tumor cell surface antigens (such as HER-2, CD79b, etc) through antibodies, internalizing them into cells, and releasing toxins (such as microtubule inhibitor: monomethyl auristatin E (MMAE) or topoisomerase inhibitor SN-38).^6^ Compared with traditional chemotherapy, ADCs can reduce damage to normal tissues and achieve more efficient treatment by killing adjacent tumor cells through the “bystander effect”.^7^ At present, ADC has been approved for application in several cancer species. For example, HER-2-targeted trastuzumab emtansine is used in breast cancer,^8^, while CD79b-targeted polatuzumab vedotin is approved for lymphoma.^10^^,^^11^

Trophoblast cell surface antigen 2 (Trop2), a transmembrane glycoprotein overexpressed in various cancers (e.g., triple-negative breast cancer [TNBC]^12^, non-small cell lung cancer^13^, urothelial carcinoma [UC]^14^), promotes tumor proliferation, metastasis, and drug resistance via ERK^15^, PI3K/AKT^16^, and other signaling pathways^17^, correlating with poor prognosis.^18^ Currently, Trop2-targeting ADCs such as sacituzumab govitecan are approved for TNBC^19^ and UC^20^, significantly improving survival, while novel ADCs like datopotamab deruxtecan show promise in other malignancies.^21^ Beyond direct cytotoxicity, Trop2-ADCs induce tumor cell apoptosis, releasing tumor-associated antigens to activate antigen-presenting cells and enhance T-cell-mediated immunity. Moreover, the cytotoxic payloads (e.g., SN-38) delivered by ADCs can induce immunogenic cell death, triggering the release of damage-associated molecular patterns such as ATP and HMGB1. This process enhances dendritic cell maturation and antigen cross-presentation, ultimately leading to increased infiltration of cytotoxic T lymphocytes (CD8^+^T cells) within the tumor microenvironment while reducing immunosuppressive cell populations (e.g., regulatory T cells or myeloid-derived suppressor cells).^22^^,^^23^ Furthermore, the Trop2 signaling pathway may intrinsically regulate PD-L1 expression, suggesting potential synergistic enhancement of antitumor immune responses when combined with immune checkpoint inhibitors (ICIs, e.g., anti-PD-1/PD-L1 antibodies).^24^ This mechanistic insight provides a strong theoretical foundation for developing combination therapies integrating Trop2-targeted agents with immunotherapies. With advancing understanding of Trop2 biology and continuous innovations in ADC technology, these targeted therapeutics hold significant promise for delivering precise and highly effective treatment options to broader cancer patient populations.

However, the expression patterns and clinical relevance of Trop2 in digestive cancers remain unclear. This study analyzed Trop2 expression and its association with clinicopathological features (e.g., stage, differentiation, metastasis, etc) in 2370 digestive cancer patients, aiming to identify key populations that may benefit from Trop2-ADC therapy and guide future precision treatment strategies.

## Materials and methods

### Patient inclusion and sample collection

Clinical and pathological data from 2370 patients with histologically confirmed digestive system tumors, comprising 804 gastric cancer (GC), 1384 colorectal cancer (CRC), and 182 PC cases, were retrospectively collected at Tongji Hospital, Huazhong University of Science and Technology between June 2022 and June 2024. All patients had available formalin-fixed, paraffin-embedded (FFPE) tumor tissue samples. Trop2 expression, routinely assessed in GC, CRC, and PC cases at our institution, was detected by immunohistochemical (IHC) staining. This study was approved by the Ethics Committee of Tongji Hospital, Huazhong University of Science and Technology (Approval No. TJ-IRB20201221) and conducted in accordance with the ethical standards outlined in the Declaration of Helsinki.

Additionally, clinical data, including clinicopathological characteristics (gender, age, tumor site, histological type, differentiation grade, TNM staging was determined according to the treatment pathway: pathological staging was applied for patients who underwent surgical resection, whereas clinical staging was used for those who did not receive surgery, metastasis status, signet ring cell carcinoma [SRCC] components, mucinous adenocarcinoma [MAC] components, vascular invasion [VI], periurinal invasion [PNI], etc.) and molecular biological characteristics (mismatch repair [MMR] status, PD-L1 CPS, HER-2 expression [confirmed by fluorescence in situ hybridization/next-generation sequencing [NGS] if IHC > 2+], KI-67 index [assessed by manual counting], and KRAS/NRAS/BRAF [KNB] mutations [assessed by NGS]), were retrospectively collected. The staging was performed according to the AJCC TNM Staging System, 8th Edition (2017). Patients with complete clinicopathological and molecular data were included, while those with incomplete records were excluded.

### IHC staining

Tissue sections were subjected to IHC staining using a Trop2-specific monoclonal antibody (Maxim, MXR033, clone A1, China) as the primary antibody, diluted 1:200 in phosphate-buffered saline. The sections were incubated with the primary antibody overnight at 4 °C. The next day, slides were incubated with a horseradish peroxidase-conjugated secondary antibody (diluted 1:500) for 1 hour at room temperature. Visualization was achieved using 3,3′-diaminobenzidine as the chromogenic substrate. Counterstaining was performed with hematoxylin to highlight cell nuclei, followed by mounting with standard mounting medium. This optimized protocol ensures consistent and reproducible detection of Trop2 expression across tissue samples.

### Trop2 classification

**Figure 1.**
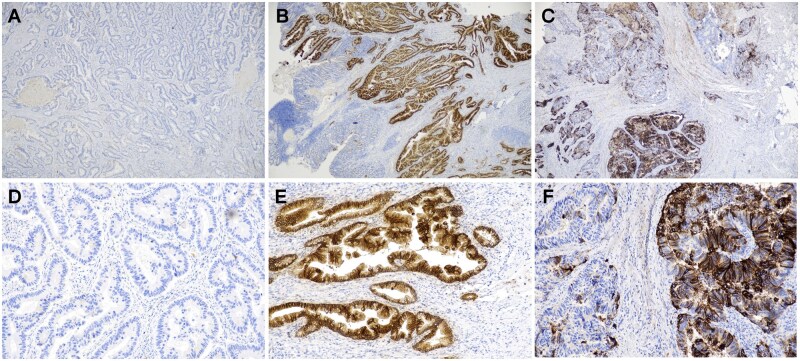
Histological and immunohistochemical (IHC) images of Trop2 expression in different levels. (A-C) Low-magnification (×4) and (D-F) high-magnification (×200) views of Trop2 IHC staining. (A, D) Trop2 negative expression. (B, E) Trop2 low expression. (C, F) Trop2 high expression.

Trop2 expression was determined by IHC as previously described^25^ and independently evaluated by 2 experienced pathologists who were blinded to the clinical data. Staining intensity was scored on a 4-point scale: 0 (negative), 1+ (weak), 2+ (moderate), or 3+ (strong), and the percentage of positive cells was categorized as follows: 0 (0%), 1 (1%-25%), 2 (26%-50%), 3 (51%-75%), or 4 (76%-100%), based on manual counting. A combined scoring system similar to the H-score was used to quantify Trop2 expression by multiplying the intensity score by the percentage score, yielding a total score ranging from 0 to 12. The distribution of total scores showed a bimodal pattern with a nadir around 4-5. Based on this, Trop2 expression was categorized as follows: negative (score 0), low expression (score 1-4), and high expression (score ≥5), consistent with previously published criteria.^25^ Histological and IHC images depicting Trop2 expression at various levels are shown in Figure 1. In addition, Trop2 intensity-based positivity was defined as moderate-to-strong staining (intensity grade ≥ 2), which was classified as positive, while otherwise was classified as negative. This was used for univariate and multivariate logistic regression.

### Statistical analysis

Data were analyzed using R software (version 4.1.2). Univariate and multivariate logistic regression analyses were used to evaluate the correlation between various clinicopathological and molecular pathological indicators and Trop2 expression. Statistical significance was defined as follows: *P* < 0.05, ^*^P < 0.01, ^**^P < 0.001, ^***^P < 0.0001, with “ns” indicating no significant difference.

## Results

### Patient characteristics

A total of 2370 patients with digestive tract tumors were included in this study, comprising 804 cases of GC (33.92%), 1384 cases of CRC (58.40%), and 182 cases of PC (7.68%). Among all patients, 62.95% (1492/2370) were male, and 1024 patients (43.21%) were over 65 years old. Regarding tumor location, among GC patients (*n *= 804), 19 cases (2.36%) were located at the gastroesophageal junction, 147 cases (18.28%) at the cardia, 187 cases (23.26%) at the gastric body, 24 cases (2.99%) at the gastric fundus, 291 cases (36.19%) at the gastric antrum, 108 cases (13.43%) at the lesser curvature, 8 cases (1.00%) at the pylorus, and 20 cases (2.49%) at the gastric remnant. According to the Lauren classification (*n *= 671), 171 cases (25.48%) were of the intestinal type, 169 cases (25.19%) of the diffuse type, 299 cases (44.56%) of the mixed type, and 32 cases (4.77%) of the indeterminate type. Among CRC patients (*n *= 1384), 498 cases (35.98%) were located in the colon, 228 cases (16.47%) in the sigmoid colon, 648 cases (46.82%) in the rectum, and 10 cases (0.72%) in the cecum. The majority (2344, 98.90%) of patients had adenocarcinoma as their histological type, with 183 cases (7.72%) containing SRCC components and 216 cases (9.11%) containing MAC components. In terms of differentiation, poorly differentiated cases were the most common, accounting for 1313 cases (55.40%), followed by moderately differentiation in 850 cases (35.86%), and well differentiation in 207 cases (8.73%). For TNM staging, T3 (48.10%, 1140 cases), N0 (45.53%, 1079 cases), and M0 (83.88%, 1988 cases) were the most common stages. Distant metastasis was observed in 307 patients (12.95%). According to TNM staging, 484 patients (20.42%) were in stage I, 646 patients (27.26%) in stage II, 858 patients (36.20%) in stage III, and 307 patients (12.95%) in stage IV. Pathological results revealed VI in 37.36% patients (788/2109) and PNI in 50.36% patients (1062/2109). Molecular pathological diagnosis showed that 94.81% (*n *= 2247) of patients had proficient MMR (pMMR), while 5.19% (*n *= 123) had deficient MMR (dMMR). Further analysis of dMMR patients for tumor MMR gene defects identified 23 cases (18.70%) with MSH2 defects, 83 cases (67.48%) with MLH1 defects, 27 cases (21.95%) with MSH6 defects, and 94 cases (76.42%) with PMS2 defects. Additionally, 61.76% patients (21/34) were PD-L1 CPS positive, 2.97% patients (66/2220) were HER-2 positive, and 95.08% patients (2241/2357) had KI-67 expression greater than 25%. Among 853 patients tested for KRAS, NRAS, and BRAF mutations, 404 patients had KRAS ­mutations, 27 patients had NRAS mutations, and 30 patients had BRAF mutations. The clinicopathological characteristics of each cancer type are summarized in Table 1.

**Table 1.** The clinicopathological features of all cases (*n *= 2370), gastric cancer (*n *= 804), colorectal cancer (*n *= 1384), and pancreatic cancer (*n *= 182).

Characteristics	All cases (*n *= 2370)	Gastric cancer (*n *= 804)	Colorectal cancer (*n *= 1384)	Pancreatic cancer (*n *= 182)
	Cases (%)	Cases (%)	Cases (%)	Cases (%)
Gender				
Male	1492 (62.95)	561 (69.78)	818 (59.10)	113 (62.09)
Female	878 (37.05)	243 (30.22)	566 (40.90)	69 (37.91)
Age (years)				
<65	1346 (56.79)	437 (54.35)	797 (57.59)	112 (61.54)
≥65	1024 (43.21)	367 (45.65)	587 (42.41)	70 (38.46)
Tumor sites				
Stomach	804 (33.92)	804 (100.00)	/	/
Gastroesophageal junction		19 (2.36)		
Cardia		147 (18.28)		
Gastric body		187 (23.26)		
Gastric fundus		24 (2.99)		
Gastric antrum		291 (36.19)		
Lesser curvature		108 (13.43)		
Pylorus		8 (1.00)		
Gastric remnant		20 (2.49)		
Colorectum	1384 (58.40)	/	1384 (100.00)	/
Colon			498 (35.98)	
Sigmoid colon			228 (16.47)	
Rectum			648 (46.82)	
Cecum			10 (0.72)	
Pancreas	182 (7.68)	/	/	182 (100.00)
Histological classification				
Adenocarcinoma	2344 (98.90)	789 (98.13)	1378 (99.57)	177 (97.25)
Others	26 (1.10)	15 (1.87)	6 (0.43)	5 (2.75)
Signet ring cell carcinoma components (SRCC)				
Yes	183 (7.72)	158 (19.65)	23 (1.66)	2 (1.10)
No	2187 (92.28)	646 (80.35)	1361 (98.34)	180 (98.90)
Mucinous adenocarcinoma components (MAC)				
Yes	216 (9.11)	34 (4.23)	176 (12.72)	6 (3.30)
No	2154 (90.89)	770 (95.77)	1208 (87.28)	176 (96.70)
Lauren classification (*n *= 671, 671, 0, 0)				
Intestinal type	171 (25.48)	171 (25.48)	/	/
Diffuse type	169 (25.19)	169 (25.19)	/	/
Mixed type	299 (44.56)	299 (44.56)	/	/
Indeterminate type	32 (4.77)	32 (4.77)	/	/
Differentiation degree				
Poor	1313 (55.40)	639 (79.48)	557 (40.25)	117 (64.29)
Moderate	850 (35.86)	91 (11.32)	714 (51.59)	45 (24.73)
Well	207 (8.73)	74 (9.20)	113 (8.16)	20 (10.99)
T stage				
1	287 (12.11)	189 (23.51)	71 (5.13)	27 (14.84)
2	355 (14.98)	90 (11.19)	170 (12.28)	95 (52.20)
3	1140 (48.10)	269 (33.46)	842 (60.84)	29 (15.93)
4	398 (16.79)	190 (23.63)	190 (13.73)	18 (9.89)
NA	190 (8.02)	66 (8.21)	111 (8.02)	13 (2.20)
N stage				
0	1079 (45.53)	337 (41.92)	646 (46.69)	96 (52.75)
1	548 (23.12)	119 (14.80)	371 (26.81)	58 (31.87)
2	388 (16.37)	117 (14.55)	256 (18.50)	15 (8.24)
3	165 (6.96)	165 (20.52)	/	/
NA	190 (8.02)	66 (8.21)	111 (8.02)	4 (2.20)
M stage				
0	1988 (83.88)	698 (86.82)	1153 (83.31)	137 (75.27)
1	307 (12.95)	78 (9.70)	188 (13.58)	41 (22.53)
NA	75 (3.16)	28 (3.48)	43 (3.11)	4 (2.20)
Stage				
I	484 (20.42)	229 (28.48)	189 (13.66)	66 (36.26)
II	646 (27.26)	170 (21.14)	433 (31.29)	43 (23.63)
III	858 (36.20)	299 (37.19)	531 (38.37)	28 (15.93)
IV	307 (12.95)	78 (9.70)	188 (13.58)	41 (22.53)
NA	75 (3.16)	28 (3.48)	43 (3.11)	4 (2.20)
Metastasis				
Yes	307 (12.95)	78 (9.70)	188 (13.58)	41 (22.53)
No	2063 (87.05)	726 (90.30)	1196 (86.42)	141 (77.47)
Vascular invasion (VI, *n *= 2109, 723, 1256, 130)				
Yes	788 (37.36)	340 (47.03)	407 (32.40)	41 (31.54)
No	1321 (62.64)	383 (52.97)	849 (67.60)	89 (68.46)
Perineural invasion (PNI, *n *= 2109, 723, 1256, 130)				
Yes	1062 (50.36)	386 (53.39)	564 (44.90)	112 (86.15)
No	1047 (49.64)	337 (46.61)	692 (55.10)	18 (13.85)
Mismatch repair status (MMR)				
Proficient MMR	2247 (94.81)	765 (95.15)	1301 (94.00)	181 (99.45)
Deficient MMR	123 (5.19)	39 (5.85)	83 (6.00)	1 (0.55)
Functional defects of tumor mismatch repair genes (*n *= 123, 39, 83, 1)				
MSH2	23 (18.70)	0 (0.00)	23 (27.71)	0 (0.00)
MLH1	83 (67.48)	35 (89.74)	48 (57.83)	0 (0.00)
MSH6	27 (21.95)	0 (0.00)	26 (31.33)	1 (100.00)
PMS2	94 (76.42)	38 (97.44)	56 (67.47)	0 (0.00)
PD-L1 CPS (*n *= 34, 27, 5, 2)				
Negative	13 (38.24)	9 (33.33)	3 (60.00)	1 (50.00)
Positive	21 (61.76)	18 (66.67)	2 (40.00)	1 (50.00)
HER-2 (*n *= 2220, 802, 1371, 47)				
Negative	2154 (97.03)	755 (94.14)	1352 (98.61)	47 (100.00)
Positive	66 (2.97)	47 (5.86)	19 (1.39)	0 (0.00)
KI-67 (*n *= 2357, 799, 1377, 181)				
<5%	28 (1.19)	8 (1.00)	9 (0.65)	11 (6.08)
5%-25%	88 (3.73)	27 (3.38)	11 (0.80)	50 (27.62)
>25%	2241 (95.08)	764 (95.62)	1357 (98.55)	120 (66.30)
KNB Mutations (*n *= 853, 13, 836, 4)				
KRAS				
Mutation	404 (47.36)	1 (7.69)	401 (47.97)	2 (50.00)
Negative	449 (52.64)	12 (92.31)	435 (52.03)	2 (50.00)
NRAS				
Mutation	27 (3.17)	0 (0.00)	27 (3.23)	0 (0.00)
Negative	826 (96.83)	13 (100.00)	809 (96.77)	4 (100.00)
BRAF				
Mutation	30 (3.52)	2 (15.38)	28 (3.35)	0 (0.00)
Negative	823 (96.48)	11 (84.62)	808 (96.65)	4 (100.00)
Trop2				
-	540 (22.78)	58 (7.21)	475 (34.32)	7 (3.85)
+	1830 (77.22)	746 (92.79)	909 (65.68)	175 (96.15)
Score (*n *= 2370, 804, 1384, 182)				
Negative	540 (22.78)	58 (7.21)	475 (34.32)	7 (3.85)
Low	1375 (58.02)	528 (65.67)	744 (53.76)	103 (56.59)
High	455 (19.20)	218 (27.11)	165 (11.92)	72 (39.56)
In Trop2+				
Regional area (*n *= 1830, 746, 909, 175)				
<25%	602 (32.90)	166 (22.25)	432 (47.52)	4 (2.29)
25%-50%	603 (32.95)	282 (37.80)	265 (29.15)	56 (32.00)
50%-75%	232 (12.68)	113 (15.15)	95 (10.45)	24 (13.71)
>75%	393 (21.48)	185 (24.80)	117 (12.87)	91 (52.00)
Intensity (*n *= 1830, 746, 909, 175)				
Weak	1198 (65.46)	488 (65.42)	610 (67.11)	100 (57.14)
Medium	186 (10.16)	72 (9.65)	103 (11.33)	11 (6.29)
Strong	446 (24.37)	186 (24.93)	196 (21.56)	64 (36.57)

### Trop2 expression characteristics

#### All tumor type

Among 2370 patients, 77.22% (1830/2370) exhibited Trop2 expression. Specifically, 602 cases (32.90%) had an expression area of less than 25%, 603 cases (32.95%) had an expression area between 25% and 50%, 232 cases (12.68%) had an expression area between 50% and 75%, while 393 cases (21.48%) had an expression area exceeding 75%. In addition, 1198 patients (65.45%) demonstrated weak Trop2 expression intensity, 186 patients (10.16%) showed medium intensity, and 446 patients (24.37%) exhibited strong intensity. Furthermore, the Trop2 score was calculated using the product of staining area and intensity. Among the samples, 22.78% (540/2370) were Trop2-negative (score = 0), 58.02% (1375/2370) were Trop2-low (score = 1-4), and 19.20% (455/2370) were Trop2-high (score ≥ 5) (Table 1).

From a clinicopathological perspective, Trop2 expression was broadly observed in males, though its intensity was comparable between genders, and the Trop2 score was high in males. In individuals older than 65 years, Trop2 expression and score were slightly lower than in younger patients, yet their positive expression area and intensity were higher. Regarding tumor types, PC had the highest proportion of Trop2-expressing tumors (96.15%), with the largest positive expression area (52%), the highest intensity (36.57%), and the highest score (39.56%). Tumors with SRCC components showed significantly higher Trop2 expression levels, larger expression areas, greater intensity, and Trop2 score than non-SRCC tumors. Similarly, tumors with MAC components exhibited higher Trop2 expression, intensity, and score but had a smaller positive area compared to non-MAC tumors. Moreover, patients with VI and PNI demonstrated significantly increased Trop2 expression, expression area, intensity, and score compared to noninvasive tumors. In terms of tumor staging, stage IV tumors had markedly higher Trop2 expression, larger expression areas, greater intensity, and higher score than non-stage IV tumors. Within the TNM staging system, T1 tumors had the highest Trop2 expression, while T4 tumors exhibited the largest expression area, greatest intensity, and highest score. Additionally, N3 and M1 tumors exhibited the highest expression levels, largest areas, greatest intensity, and highest score. Poorly differentiated tumors had the highest Trop2 expression and score, whereas well differentiated tumors displayed the largest expression area and greatest intensity (Table 2 and Table S1).

**Table 2.** Expression of Trop2 among different clinicopathological features: classification based on Trop2 score.

Characteristics	All cases (*n *= 2370)			Gastric cancer (*n *= 804)			Colorectal cancer (*n *= 1384)			Pancreatic cancer (*n *= 182)		
	Negative (%)	Low (%)	High (%)	Negative (%)	Low (%)	High (%)	Negative (%)	Low (%)	High (%)	Negative (%)	Low (%)	High (%)
Gender	*n *= 540	*n *= 1375	*n *= 455	*n *= 58	*n *= 528	*n *= 218	*n *= 475	*n *= 744	*n *= 165	*n *= 7	*n *= 103	*n *= 72
Male	319 (59.07)	882 (64.15)	291 (63.96)	29 (50.00)	375 (71.02)	157 (72.02)	284 (59.79)	437 (58.74)	97 (58.79)	6 (85.71)	70 (67.96)	37 (51.39)
Female	221 (40.93)	493 (35.85)	164 (36.04)	29 (50.00)	153 (28.98)	61 (27.98)	191 (40.21)	307 (41.26)	68 (41.21)	1 (14.29)	33 (32.04)	35 (48.61)
Age (years)												
<65	291 (53.89)	793 (57.67)	262 (57.58)	29 (50.00)	304 (57.58)	104 (47.71)	259 (54.53)	428 (57.53)	110 (66.67)	3 (42.86)	61 (59.22)	48 (66.67)
≥65	249 (46.11)	582 (42.33)	193 (42.42)	29 (50.00)	224 (42.42)	114 (52.29)	216 (45.47)	316 (42.47)	55 (33.33)	4 (57.14)	42 (40.78)	24 (33.33)
Tumor sites												
Stomach	58 (10.74)	528 (38.40)	218 (47.91)	58 (10.74)	528 (38.4)	218 (47.91)						
Gastroesophageal junction				1 (1.72)	13 (2.46)	5 (2.29)						
Cardia				7 (12.07)	92 (17.42)	48 (22.02)						
Gastric body				13 (22.41)	125 (23.67)	49 (22.48)						
Gastric fundus				1 (1.72)	18 (3.41)	5 (2.29)						
Gastric antrum				24 (41.38)	200 (37.88)	67 (30.73)						
Lesser curvature				9 (15.52)	64 (12.12)	35 (16.06)						
Pylorus				2 (3.45)	4 (0.76)	2 (0.92)						
Gastric remnant				1 (1.72)	12 (2.27)	7 (3.21)						
Colorectum	475 (87.96)	744 (54.11)	165 (36.26)				475 (87.96)	744 (54.11)	165 (36.26)			
Colon							179 (37.68)	260 (34.95)	59 (35.76)			
Sigmoid colon							91 (19.16)	115 (15.46)	22 (13.33)			
Rectum							202 (42.53)	364 (48.92)	82 (49.70)			
Cecum							3 (0.63)	5 (0.67)	2 (1.21)			
Pancreas	7 (1.30)	103 (7.49)	72 (15.82)							7 (1.3)	103 (7.49)	72 (15.82)
Histological classification												
Adenocarcinoma	533 (98.70)	1361 (98.98)	450 (98.90)	57 (98.28)	519 (98.30)	213 (97.71)	471 (99.16)	742 (99.73)	165 (100.00)	5 (71.43)	100 (97.09)	72 (100.00)
Others	7 (1.30)	14 (1.02)	5 (1.10)	1 (1.72)	9 (1.70)	5 (2.29)	4 (0.84)	2 (0.27)	0 (0.00)	2 (28.57)	3 (2.91)	0 (0.00)
Signet ring cell carcinoma components (SRCC)												
Yes	15 (2.78)	117 (8.51)	51 (11.21)	10 (17.24)	102 (19.32)	46 (21.10)	5 (1.05)	14 (1.88)	4 (2.42)	0 (0.00)	1 (0.97)	1 (1.39)
No	525 (97.22)	1258 (91.49)	404 (88.79)	48 (82.76)	426 (80.68)	172 (78.90)	470 (98.95)	730 (98.12)	161 (97.58)	7 (100.00)	102 (99.03)	71 (98.61)
Mucinous adenocarcinoma components (MAC)												
Yes	51 (9.44)	123 (8.95)	42 (9.23)	1 (1.72)	23 (4.36)	10 (4.59)	50 (10.53)	97 (13.04)	29 (17.58)	0 (0.00)	3 (2.91)	3 (4.17)
No	489 (90.56)	1252 (91.05)	413 (90.77)	57 (98.28)	505 (95.64)	208 (95.41)	425 (89.47)	647 (86.96)	136 (82.42)	7 (100.00)	100 (97.09)	69 (95.83)
Lauren classification (*n *= 671, 671, 0, 0)	*n *= 48	*n *= 442	*n *= 181	*n *= 48	*n *= 442	*n *= 181	/	/	/	/	/	/
Intestinal type	14 (29.17)	104 (23.53)	53 (29.28)	14 (29.17)	104 (23.53)	53 (29.28)						
Diffuse type	18 (37.50)	121 (27.38)	30 (16.57)	18 (37.50)	121 (27.38)	30 (13.76)						
Mixed type	15 (31.25)	192 (43.44)	92 (50.83)	15 (31.25)	192 (43.44)	92 (42.20)						
Indeterminate type	1 (2.08)	25 (5.66)	6 (3.31)	1 (2.08)	25 (5.66)	6 (2.75)						
Differentiation degree												
Poor	220 (40.74)	790 (57.45)	303 (66.59)	44 (75.86)	427 (80.87)	168 (77.06)	170 (35.79)	300 (40.32)	87 (52.73)	6 (85.71)	63 (61.17)	48 (66.67)
Moderate	268 (49.63)	468 (34.04)	114 (25.05)	10 (17.24)	51 (9.66)	30 (13.76)	257 (54.11)	388 (52.15)	69 (41.82)	1 (14.29)	29 (28.16)	15 (20.83)
Well	52 (9.63)	117 (8.51)	38 (8.35)	4 (6.90)	50 (9.47)	20 (9.17)	48 (10.11)	56 (7.53)	9 (5.45)	0 (0.00)	11 (10.68)	9 (12.50)
T stage												
1	42 (7.78)	189 (13.75)	56 (12.31)	17 (29.31)	135 (25.57)	37 (16.97)	25 (5.26)	41 (5.51)	5 (3.03)	0 (0.00)	13 (12.62)	14 (19.44)
2	79 (14.63)	197 (14.33)	79 (17.36)	8 (13.79)	55 (10.42)	27 (12.39)	69 (14.53)	86 (11.56)	15 (9.09)	2 (28.57)	56 (54.37)	37 (51.39)
3	316 (58.52)	643 (46.76)	181 (39.78)	19 (32.76)	173 (32.77)	77 (35.32)	295 (62.11)	455 (61.16)	92 (55.76)	2 (28.57)	15 (14.56)	12 (16.67)
4	68 (12.59)	24 (1.75)	89 (19.56)	10 (17.24)	126 (23.86)	54 (24.77)	56 (11.79)	102 (13.71)	32 (19.39)	2 (28.57)	13 (12.62)	3 (4.17)
NA	35 (6.48)	105 (7.64)	50 (10.99)	4 (6.90)	39 (7.39)	23 (10.55)	30 (6.32)	60 (8.06)	21 (12.73)	1 (14.29)	6 (5.83)	6 (8.33)
N stage												
0	299 (55.37)	603 (43.85)	177 (38.90)	29 (50.00)	222 (42.05)	86 (39.45)	265 (55.79)	323 (43.41)	58 (35.15)	5 (71.43)	58 (56.31)	33 (45.83)
1	128 (23.70)	318 (23.13)	102 (22.42)	9 (15.52)	79 (14.96)	31 (14.22)	119 (25.05)	207 (27.82)	45 (27.27)	0 (0.00)	32 (31.07)	26 (36.11)
2	66 (12.22)	235 (17.09)	88 (19.34)	4 (6.90)	74 (14.02)	40 (18.35)	61 (12.84)	154 (20.7)	41 (24.85)	1 (14.29)	7 (6.8)	7 (9.72)
3	12 (2.22)	114 (8.29)	38 (8.35)	12 (20.69)	114 (21.59)	38 (17.43)	/	/	/	/	/	/
NA	35 (6.48)	105 (7.64)	56 (12.31)	4 (6.90)	39 (7.39)	23 (10.55)	30 (6.32)	60 (8.06)	21 (12.73)	1 (14.29)	6 (5.83)	6 (8.33)
M stage												
0	465 (86.11)	1154 (83.93)	369 (81.10)	53 (91.38)	460 (87.12)	185 (84.86)	407 (85.68)	614 (82.53)	132 (80.00)	5 (71.43)	80 (77.67)	52 (72.22)
1	62 (11.48)	172 (12.51)	73 (16.04)	4 (6.90)	47 (8.90)	27 (12.39)	56 (11.79)	104 (13.98)	28 (16.97)	2 (28.57)	21 (20.39)	18 (25.00)
NA	13 (2.41)	49 (3.56)	13 (2.86)	1 (1.72)	21 (3.98)	6 (2.75)	12 (2.53)	26 (3.49)	5 (3.03)	0 (0.00)	2 (1.94)	2 (2.78)
Stage												
I	102 (18.89)	297 (21.6)	85 (18.68)	21 (36.21)	161 (30.49)	47 (21.56)	79 (16.63)	97 (13.04)	13 (7.88)	2 (28.57)	39 (37.86)	25 (34.72)
II	190 (35.19)	336 (24.44)	120 (26.37)	15 (25.86)	99 (18.75)	56 (25.69)	175 (36.84)	214 (28.76)	44 (26.67)	0 (0.00)	23 (22.33)	20 (27.78)
III	173 (32.04)	521 (37.89)	164 (36.04)	17 (29.31)	200 (37.88)	82 (37.61)	153 (32.21)	303 (40.73)	75 (45.45)	3 (42.86)	18 (17.48)	7 (9.72)
IV	62 (11.48)	172 (12.51)	73 (16.04)	4 (6.90)	47 (8.90)	27 (12.39)	56 (11.79)	104 (13.98)	28 (16.97)	2 (28.57)	21 (20.39)	18 (25.00)
NA	13 (2.41)	49 (3.56)	13 (2.86)	1 (1.72)	21 (3.98)	6 (2.75)	12 (2.53)	26 (3.49)	5 (3.03)	0 (0.00)	2 (1.94)	2 (2.78)
Metastasis												
Yes	62 (11.48)	172 (12.51)	73 (16.04)	4 (6.90)	47 (8.90)	27 (12.39)	56 (11.79)	104 (13.98)	28 (16.97)	2 (28.57)	21 (20.39)	18 (25.00)
No	478 (88.52)	1203 (87.49)	382 (83.96)	54 (93.10)	481 (91.10)	191 (87.61)	419 (88.21)	640 (86.02)	137 (83.03)	5 (71.43)	82 (79.61)	54 (75.00)
Vascular invasion (VI, *n *= 2109, 723, 1256, 130)	*n *= 492	*n *= 1231	*n *= 380	*n *= 53	*n *= 478	*n *= 192	*n *= 434	*n *= 680	*n *= 142	*n *= 5	*n *= 73	*n *= 52
Yes	132 (26.83)	456 (37.04)	200 (52.63)	17 (32.08)	213 (44.56)	110 (57.29)	112 (25.81)	225 (33.09)	70 (49.30)	3 (60.00)	18 (24.66)	20 (38.46)
No	360 (73.17)	775 (62.96)	180 (47.37)	36 (67.92)	265 (55.44)	82 (42.71)	322 (74.19)	455 (66.91)	72 (50.70)	2 (40.00)	55 (75.34)	32 (61.54)
Perineural invasion (PNI, *n *= 2109, 723, 1256, 130)	*n *= 492	*n *= 1231	*n *= 380	*n *= 53	*n *= 478	*n *= 192	*n *= 434	*n *= 680	*n *= 142	*n *= 5	*n *= 73	*n *= 52
Yes	184 (37.4)	639 (51.91)	239 (62.89)	25 (47.17)	246 (51.46)	115 (59.90)	158 (36.41)	330 (48.53)	76 (53.52)	1 (20.00)	63 (86.3)	48 (92.31)
No	308 (62.6)	592 (48.09)	147 (38.68)	28 (52.83)	232 (48.54)	77 (40.10)	276 (63.59)	350 (51.47)	66 (46.48)	4 (80.00)	10 (13.7)	4 (7.69)
MMR status												
pMMR	496 (91.85)	1314 (95.56)	437 (96.04)	49 (84.48)	506 (95.83)	210 (96.33)	441 (92.84)	705 (94.76)	155 (93.94)	6 (85.71)	103 (100)	72 (100)
dMMR	44 (8.15)	61 (4.44)	18 (3.96)	9 (15.52)	22 (4.17)	8 (3.67)	34 (7.16)	39 (5.24)	10 (6.06)	1 (14.29)	0 (0.00)	0 (0.00)
Functional defects of tumor mismatch repair genes (*n *= 123, 39, 83, 1)	*n = *44	*n = *61	*n = *18	*n = *9	*n = *22	*n = *8	*n = *34	*n = *39	*n = *10	*n = *1		
MSH2	12 (27.27)	8 (13.11)	3 (16.67)	0 (0.00)	0 (0.00)	0 (0.00)	12 (35.29)	8 (20.51)	3 (30.00)	0 (0.00)		
MLH1	25 (56.82)	43 (70.49)	15 (83.33)	7 (77.78)	20 (90.91)	8 (100.00)	18 (52.94)	23 (58.97)	7 (70.00)	0 (0.00)		
MSH6	14 (31.82)	11 (18.03)	2 (11.11)	0 (0.00)	0 (0.00)	0 (0.00)	13 (38.24%)	11 (28.21%)	2 (20.00%)	1 (100.00%)		
PMS2	31 (70.45%)	48 (78.69%)	15 (83.33%)	9 (100.00%)	21 (95.45%)	8 (100.00)	22 (64.71%)	27 (69.23%)	7 (70.00%)	0 (0.00%)		
PD-L1 CPS (*n *= 34, 27, 5, 2)		*n = *22	*n = *12	*n = *58	*n = 17*	*n = *10	/	*n = *3	*n = *2		*n *= 2	
Negative		8 (36.36)	5 (41.67)	0 (0.00)	6 (35.29)	3 (30.00)	/	1 (33.33)	2 (100.00)		1 (50.00)	
Positive		14 (63.64)	7 (58.33)	58 (100.00)	11 (64.71)	7 (70.00)	/	2 (66.67)	0 (0.00)		1 (50.00)	
HER-2 (*n *= 2220, 802, 1371, 47)	*n *= 531	*n *= 1290	*n *= 399	*n *= 58	*n *= 527	*n *= 217	*n *= 470	*n *= 737	*n *= 170	*n *= 3	*n *= 26	*n *= 18
Negative	525 (98.87)	1249 (96.82)	380 (95.24)	55 (94.83)	498 (94.50)	202 (93.09)	467 (99.36)	725 (98.37)	166 (97.65)	3 (100.00)	26 (100.00)	18 (100.00)
Positive	6 (1.13)	41 (3.18)	19 (4.76)	3 (5.17)	29 (5.50)	15 (6.91)	3 (0.64)	12 (1.63)	4 (2.35)	0 (0.00)	0 (0.00)	0 (0.00)
KI-67 (*n *= 2357, 799, 1377, 181)	*n = *537	*n = *1370	*n = *450	*n = *57	*n = *525	*n = *217	*n = *473	*n = *742	*n = *162	*n = *7	*n = *193	*n = *71
<5%	7 (1.30)	17 (1.24)	4 (0.89)	2 (3.51)	5 (0.95)	1 (0.46)	3 (0.63)	6 (0.81)	0 (0.00)	2 (28.57)	6 (3.11)	3 (4.23)
5%-25%	9 (1.68)	56 (4.09)	23 (5.11)	5 (8.77)	17 (3.24)	5 (2.30)	4 (0.85)	6 (0.81)	1 (0.62)	0 (0.00)	33 (17.10)	17 (23.94)
>25%	521 (97.02)	1297 (94.67)	423 (94.00)	50 (87.72)	503 (95.81)	211 (97.24)	466 (98.52)	730 (98.38)	161 (99.38)	5 (71.43)	64 (33.16)	51 (71.83)
KNB Mutations (*n *= 853, 13, 836, 4)												
KRAS	*n *= 291	*n *= 460	*n *= 102	*n *= 0	*n *= 7	*n *= 6	*n *= 290	*n *= 452	*n *= 94	*n *= 1	*n *= 1	*n *= 2
Mutation	112 (38.49)	238 (51.74)	54 (52.94)		0 (0.00)	1 (16.67)	112 (38.62)	238 (52.65)	51 (54.26)	0 (0.00)	0 (0.00)	2 (100.00)
Negative	179 (61.51)	222 (48.26)	48 (47.06)		7 (100.00)	5 (83.33)	178 (61.38)	214 (47.35)	43 (45.74)	1 (100.00)	1 (100.00)	0 (0.00)
NRAS												
Mutation	9 (3.09)	16 (3.48)	2 (1.96)		0 (0.00)	0 (0.00)	9 (3.10)	16 (3.54)	2 (2.13)	0 (0.00)	0 (0.00)	0 (0.00)
Negative	282 (96.91)	444 (96.52)	100 (98.04)		7 (100.00)	6 (100.00)	281 (96.90)	436 (96.46)	92 (97.87)	1 (100.00)	1 (100.00)	2 (100.00)
BRAF												
Mutation	8 (2.75)	19 (4.13)	3 (2.94)		1 (14.29)	1 (16.67)	8 (2.76)	18 (3.98)	2 (2.13)	0 (0.00)	0 (0.00)	0 (0.00)
Negative	283 (97.25)	441 (95.87)	99 (97.06)		6 (85.71)	5 (83.33)	282 (97.24)	434 (96.02)	92 (97.87)	1 (100.00)	1 (100.00)	2 (100.00)

In the analysis of tumor molecular pathological features, Trop2 expression and score were significantly higher in pMMR patients compared to dMMR patients. Among MMR protein deficiencies, MLH1-deficient patients exhibited the highest Trop2 expression, intensity, and score, while MSH2-deficient patients had the largest expression area. Patients with HER-2 positivity and PD-L1 CPS negativity showed the highest Trop2 expression, expression area, intensity, and score. Regarding KI-67 expression, those with 5%-25% expression displayed the highest Trop2 expression, largest positive expression area and highest score, while patients with KI-67 expression above 25% had the highest Trop2 intensity. Finally, genetic analysis of KRAS, NRAS, and BRAF revealed that Trop2 expression and score were significantly elevated in KNB-mutated (Mut) patients compared to wild-type (WT), though the expression area and intensity remained similar, see Table 2 and Table S1.

#### Gastric cancer

**Figure 2.**
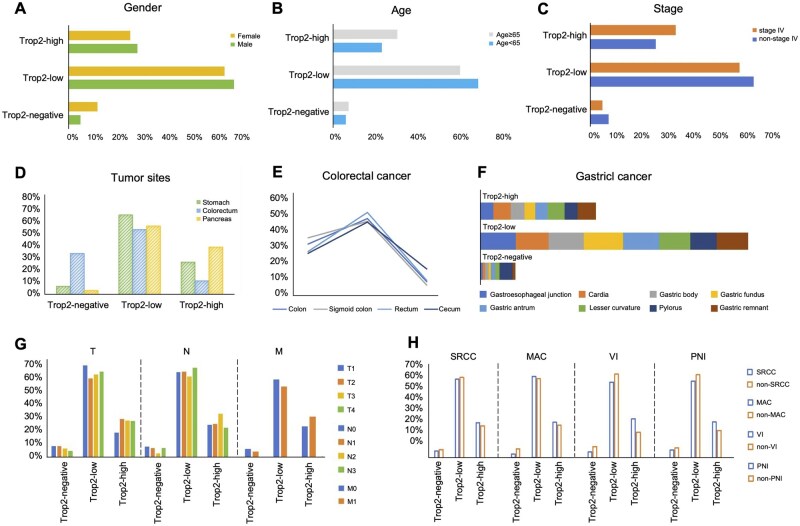
Trop2 expression in relation to clinicopathological and staging across tumor types. (A-B) Trop2 expression in different genders (A) and age groups (B). (C) Trop2 expression according to tumor stage. (D-F) Trop2 expression by tumor location: (D) all sites, (E) colorectal cancer (CRC), and (F) gastric cancer (GC). (G) Trop2 expression across TNM stages. (H) Trop2 expression by histological subtypes, including presence or absence of signet ring cell carcinoma (SRCC), mucinous adenocarcinoma (MAC), and invasive features such as vascular invasion (VI) and perineural invasion (PNI).

Among 804 GC patients, 92.79% (746/804) exhibited Trop2 expression. The distribution of expression area was as follows: 22.25% (166/746) had an expression area of less than 25%, 37.8% (282/746) between 25% and 50%, 15.15% (113/746) between 50% and 75%, and 24.8% (185/746) exceeding 75%. In terms of intensity, 488 patients exhibited weak positivity, 72 moderate intensity, and 186 strong intensity (Table 1). For Trop2 score, 218 (27.11%) patients showed Trop2-high, 528 (65.67%) patients showed Trop2-low, and 58 (7.21%) patients showed Trop2-negative (Figure 2D, Table 1). In GC, males exhibited higher Trop2 expression levels, expression areas, intensities, and scores than females (Figure 2A). Patients under 65 years had higher Trop2 expression levels, but their expression areas, intensities, and scores were lower than those over 65 years (Figure 2B). Expression levels were consistent across different GC sites (Figure 2F). The expression trends for SRCC and MAC components, as well as for VI and PNI involvement, were in line with those observed across all tumor types (Figure 2H). Regarding staging and differentiation, patients classified as T4, N2, and M1 (stage IV) exhibited the highest Trop2 expression, expression areas, intensities, and scores (Figure 2G). Poorly differentiated tumors exhibited the highest Trop2 expression levels and intensities, while moderately differentiated tumors had the largest expression area, and scores (Table 2 and Table S1).

Molecularly, pMMR patients had higher Trop2 expression, expression areas, and scores but slightly lower intensity compared to dMMR patients. MLH1-deficient patients exhibited the highest Trop2 expression area, intensity, and scores. Patients with HER-2 positivity and PD-L1 CPS negativity showed the highest Trop2 expression. In terms of KI-67 expression, patients with expression above 25% exhibited the highest Trop2 levels, intensity, and scores, while those with 5%-25% expression had the largest expression area. According to the genetic testing results, no NRAS mutations were detected in GC cases. Patients with KRAS and BRAF mutations exhibited higher Trop2 expression levels, expression areas, intensities, and scores compared to WT patients (see Table 2 and Table S1).

#### Colorectal cancer

In CRC, 65.68% patients (909/1384) exhibited Trop2 expression. Of these, 47.52% (432/909) had an expression area of less than 25%, 29.15% (265/909) between 25% and 50%, 10.45% (95/909) between 50% and 75%, and 12.87% (117/909) above 75%. Weak Trop2 expression was observed in 610 patients (67.11%), moderate expression in 103 patients (11.33%), and strong expression in 196 patients (21.56%). For Trop2 score, 165 (11.92%) patients showed Trop2-high, 744 (53.76%) patients showed Trop2-low, and 475 (34.32%) patients showed Trop2-negative (Figure 2D, Table 1). Males had slightly higher Trop2 expression levels and expression areas than females, though females exhibited greater expression intensity, and scores (Table 2 and Table S1). Patients younger than 65 exhibited higher Trop2 levels, expression areas, intensities, and scores than those older than 65 (Table 2 and Table S1). Among CRC subtypes, rectal cancer had the highest Trop2 expression, expression area, intensity, and scores (Figure 2E). For tumor components, SRCC-containing tumors had significantly elevated Trop2 expression, expression areas, intensities, and scores. Mucinous adenocarcinoma-containing tumors exhibited high expression levels, areas, and scores but lower intensity than non-MAC tumors. Trends for VI and PNI involvement were consistent with those observed across all tumor types (Table 2 and Table S1). Regarding staging, patients classified as T4, N2, and M1 (stage IV) displayed the highest Trop2 expression, expression areas, intensities, and scores (Table 2 and Table S1). Moderately differentiated tumors showed the highest Trop2 expression levels and intensities, while poorly differentiated tumors showed the highest Trop2 expression areas, and scores (Table 2 and Table S1).

In the analysis of tumor molecular pathological features, Trop2 expression levels were generally higher in pMMR patients compared to dMMR patients. Specifically, in the MMR gene deficiency analysis, patients with MLH1 deficiency exhibited the highest Trop2 expression levels, intensity, and scores, whereas those with MSH2 deficiency showed the largest Trop2-positive area. Similarly, patients who were HER-2 positive and PD-L1 CPS negative demonstrated higher Trop2 expression. Regarding the relationship between KI-67 and Trop2, patients with KI-67 expression levels below 5% showed higher Trop2 expression levels; those with KI-67 expression between 5% and 25% had the largest Trop2-positive area; while patients with KI-67 expression above 25% exhibited the highest Trop2 expression intensity, and scores. Additionally, genetic testing revealed that CRC patients with KRAS, NRAS, and BRAF mutations had higher Trop2 expression levels compared to WT patients, with similar trends observed in both Trop2-positive area and expression intensity. Detailed data were provided in Table 2 and Table S1.

#### Pancreatic cancer

Among 182 PC patients, 96.15% (175/182) showed positive Trop2 expression. Specifically, 2.29% (4/175) patients had Trop2-positive areas <25%, 32% (56/175) patients between 25%-50%, 13.71% (24/175) patients between 50%-75%, and 52% (91/175) patients >75%. Regarding expression intensity, 57.14% (100/175) of patients exhibited weak positivity, 6.29% (11/175) moderate intensity, and 36.57% (64/175) strong intensity. For Trop2 score, 72 (39.56%) patients showed Trop2-high, 72 (39.56%) patients showed Trop2-low, and 7 (3.85%) patients showed Trop2-negative (Figure 2D, Table 1). The distribution of Trop2 expression across various clinicopathological features was as follows: female patients demonstrated significantly higher Trop2 expression levels, intensity, and scores compared to male patients, though their positive expression areas were slightly smaller (Table 2 and Table S1). Patients younger than 65 years exhibited higher Trop2 expression levels, positive areas, intensity, and scores compared to those older than 65 (Table 2 and Table S1). Analysis of tumor histological components revealed that patients with SRCC components had higher Trop2 expression levels, positive areas, intensity, and scores, consistent with findings in other tumors. In patients with MAC components, Trop2 expression levels, intensity, and scores were elevated, but the positive area was smaller compared to non-MAC patients. Regarding invasive features, similar to observations in other tumors, patients with VI and PNI showed higher Trop2 expression levels, larger positive areas, stronger intensity, and higher scores (Table 2 and Table S1). For tumor stage and differentiation, results differed slightly from other tumors. Non-stage IV patients had marginally higher Trop2 expression levels compared to stage IV patients (96.35% vs. 95.12%), but their positive areas, intensity, and scores were lower (Table 2 and Table S1). In TNM staging, T1, N1, and M0 stage patients had higher expression levels, while T1, N2, and M1 stage patients exhibited larger positive areas, stronger expression intensity, and higher scores (Table 2 and Table S1). In terms of differentiation, poorly differentiated patients showed higher Trop2 expression levels, larger positive areas, stronger intensity, and higher scores (Figure 2I). Detailed data were provided in Table 2 and Table S1.

Regarding molecular pathological features, due to the low number of dMMR patients and absence of Trop2 expression, MMR gene deficiency analysis could not be performed. Similarly, as all patients were HER-2 negative, the correlation between HER-2 and Trop2 expression could not be assessed. However, it was observed that PD-L1 CPS-negative patients had larger Trop2-positive areas. Additionally, a relationship between KI-67 expression and Trop2 expression was noted: patients with KI-67 < 5% showed higher Trop2 levels, those with KI-67 5%-25% had the highest expression levels, and those with KI-67 > 25% had the largest positive areas, strongest expression intensity, and highest scores. Finally, genetic testing revealed that NRAS and BRAF were WT in all PC patients. Therefore, analysis focused solely on KRAS mutations. Results indicated that patients with KRAS mutations exhibited significantly higher Trop2 expression levels, larger positive areas, stronger intensity, and higher scores compared to WT patients (Table 2 and Table S1).

### Trop2 expression differences according to clinicopathological features

Univariate and multivariate logistic regression analyses revealed that Trop2 expression status (positive vs. negative) was significantly associated with various clinicopathological factors. In all tumor types, univariate analysis revealed that Trop2 expression was significantly associated with CRC (odds ratio [OR] = 0.583 [0.480-0.709], *P* < .001), pancreatic cancer (OR = 1.483 [1.066-2.064], *P* = .019), SRCC components (OR = 0.544 [0.398-0.744], *P* < .001), moderate differentiation (OR = 0.651 [0.512-0.827], *P* < .001), VI (OR = 0.544 [0.446-0.662], *P* < .001), PNI (OR = 0.601 [0.494-0.733], *P* < .001), and MMR status (OR = 0.619 [0.389-0.983], *P* = .042); multivariate analysis further identified CRC (OR = 0.646 [0.503-0.828], *P* = .001), pancreatic cancer (OR = 1.565 [1.035-2.367], *P* = .034), SRCC components (OR = 0.514 [0.349-0.756], *P* = .001), VI (OR = 0.600 [0.481-0.749], *P* < .001), and PNI (OR = 0.778 [0.625-0.968], *P* = .024) as significant factors correlated with Trop2 expression. Specifically, in GC, multivariate analysis showed that SRCC components (OR = 0.299 [0.182-0.490], *P* < .001), Lauren classification (diffuse type: OR = 0.257 [0.143-0.460], *P* < .001; mixed type: OR = 0.537 [0.340-0.849], *P* = .008; indeterminate type: OR = 0.366 [0.143-0.937], *P* = .036) and VI (OR = 0.571 [0.379-0.859], *P* = .007) were significantly associated with Trop2 expression. In CRC, multivariate analysis identified VI (OR = 0.555 [0.414-0.743], *P* < .001) as a significant factor correlated with Trop2 expression. Finally, in pancreatic cancer, multivariate analysis revealed that gender (OR = 2.193 [1.138-4.228], *P* = .019) and T4 stage (OR = 0.156 [0.036-0.679], *P* = .013) were significantly associated with Trop2 expression (Table 3).

**Table 3.** Univariate and multivariate logistic regression analysis of risk factors in all tumor types (*n *= 2370), gastric cancer (*n *= 804), colorectal cancer (*n *= 1384), and pancreatic cancer (*n *= 182).

Variables	All cases (*n *= 2370)				Gastric cancer (*n *= 804)				Colorectal cancer (*n *= 1384)				Pancreatic cancer (*n *= 182)			
	Univariate analysis		Multivariate analysis		Univariate analysis		Multivariate analysis		Univariate analysis		Multivariate analysis		Univariate analysis		Multivariate analysis	
	Odds ratio (OR) (95% confidence interval [CI])	*P*	OR (95% CI)	*P*	OR (95% CI)	*P*	OR (95% CI)	*P*	OR (95% CI)	*P*	OR (95% CI)	*P*	OR (95% CI)	*P*	OR (95% CI)	*P*
Gender																
Male	Ref				Ref				Ref				Ref		Ref	
Female	0.990 (0.819-1.195)	0.913			0.849 (0.612-1.177)	0.326			1.049 (0.809-1.360)	0.718			2.070 (1.123-3.814)	0.020	2.193 (1.138-4.228)	0.019
Age (years)																
<65	Ref				Ref				Ref				Ref			
≥65	0.931 (0.775-1.120)	0.450			1.154 (0.857-1.553)	0.345			0.841 (0.648-1.092)	0.915			0.624 (0.336-1.158)	0.135		
Tumor sites																
Stomach	Ref		Ref		/	/			/	/			/	/		
Colorectum	0.583 (0.480-0.709)	<0.001	0.646 (0.503-0.828)	0.001												
Pancreas	1.483 (1.066-2.064)	0.019	1.565 (1.035-2.367)	0.034												
Histological classification																
Adenocarcinoma	Ref				Ref				Ref				Ref			
Others	0.823 (0.329-2.060)	0.678			1.421 (0.500-4.035)	0.510			/	0.971			/	0.988		
Signet ring cell carcinoma components (SRCC)																
Yes	Ref		Ref		Ref		Ref		Ref				Ref			
No	0.544 (0.398-0.744)	<0.001	0.514 (0.349-0.756)	0.001	0.652 (0.455-0.935)	0.020	0.299 (0.182-0.490)	<0.001	0.624 (0.254-1.531)	0.304			0.698 (0.043-11.340)	0.801		
Mucinous adenocarcinoma components (MAC)																
Yes	Ref				Ref				Ref				Ref			
No	0.851 (0.625-1.157)	0.302			0.584 (0.292-1.169)	0.129			0.749 (0.520-1.077)	0.119			0.692 (0.136-3.527)	0.658		
Lauren classification (*n *= 0, 671, 0, 0)																
Intestinal type	/	/			Ref		Ref		/	/			/	/		
Diffuse type					0.600 (0.377-0.956)	0.032	0.257 (0.143-0.460)	<0.001								
Mixed type					0.883 (0.596-1.310)	0.537	0.537 (0.340-0.849)	0.008								
Indeterminate type					0.586 (0.248-1.383)	0.223	0.366 (0.143-0.937)	0.036								
Differentiation degree																
Poor	Ref				Ref				Ref				Ref			
Moderate	0.651 (0.512-0.827)	<0.001			1.142 (0.709-1.840)	0.584			0.920 (0.583-1.453)	0.721			0.842 (0.363-1.953)	0.689		
Well	0.415 (0.171-1.012)	0.053			0.335 (0.097-1.157)	0.084			0.841 (0.224-3.157)	0.797			/	0.987		
T stage																
1	Ref				Ref				Ref				Ref		Ref	
2	1.182 (0.835-1.672)	0.345			1.887 (1.100-3.240)	0.021			0.899 (0.453-1.784)	0.761			0.557 (0.235-1.319)	0.184	0.528 (0.219-1.272)	0.155
3	0.870 (0.647-1.169)	0.356			1.537 (1.011-2.335)	0.044			0.951 (0.525-1.723)	0.869			0.565 (0.196-1.629)	0.290	0.598 (0.203-1.755)	0.349
4	1.101 (0.783-1.548)	0.581			1.542 (0.984-2.416)	0.059			1.227 (0.635-2.370)	0.542			0.160 (0.037-0.685)	0.013	0.156 (0.036-0.679)	0.013
N stage																
0	Ref				Ref				Ref				Ref			
1	1.116 (0.882-1.411)	0.361			0.945 (0.600-1.488)	0.808			1.183 (0.864-1.618)	0.294			1.702 (0.877-3.304)	0.116		
2	1.242 (0.958-1.610)	0.102			1.392 (0.901-2.152)	0.136			1.248 (0.879-1.772)	0.215			1.596 (0.533-4.780)	0.404		
3	1.126 (0.776-1.634)	0.531			0.799 (0.528-1.210)	0.289			/	/			/	/		
M stage																
0	Ref				Ref				Ref				Ref			
1	1.275 (0.981-1.657)	0.069			1.522 (0.943-2.456)	0.086			1.138 (0.789-1.641)	0.488			1.328 (0.657-2.681)	0.430		
Stage																
I	Ref				Ref				Ref				Ref			
II	1.132 (0.865-1.481)	0.365			1.748 (1.142-2.675)	0.010			1.196 (0.772-1.853)	0.423			1.469 (0.676-3.189)	0.331		
III	1.028 (0.795-1.329)	0.832			1.224 (0.835-1.794)	0.300			1.331 (0.872-2.032)	0.185			0.513 (0.191-1.377)	0.185		
IV	1.344 (0.979-1.847)	0.068			1.916 (1.119-3.281)	0.018			1.393 (0.843-2.301)	0.195			1.329 (0.604-2.921)	0.480		
Metastasis																
Yes	Ref				Ref				Ref				Ref			
No	0.785 (0.605-1.020)	0.070			0.650 (0.403-1.048)	0.077			0.887 (0.616-1.277)	0.519			0.763 (0.379-1.537)	0.449		
Vascular invasion (VI, *n *= 2109, 723, 1256, 130)																
Yes	Ref		Ref		Ref		Ref		Ref		Ref		Ref			
No	0.544 (0.446-0.662)	<0.001	0.600 (0.481-0.749)	<0.001	0.617 (0.450-0.846)	0.003	0.571 (0.379-0.859)	0.007	0.517 (0.392-0.683)	<0.001	0.555 (0.414-0.743)	<0.001	0.649 (0.308-1.370)	0.257		
Perineural invasion (PNI, *n *= 2109, 723, 1256, 130)																
Yes	Ref		Ref		Ref				Ref				Ref			
No	0.601 (0.494-0.733)	<0.001	0.778 (0.625-0.968)	0.024	0.655 (0.476-0.901)	0.009			0.674 (0.514-0.885)	0.004			0.495 (0.165-1.481)	0.208		
MMR status																
pMMR	Ref				Ref				Ref				Ref			
dMMR	0.619 (0.389-0.983)	0.042			0.622 (0.291-1.329)	0.220			0.723 (0.401-1.304)	0.281			/	0.987		
HER-2 (*n *= 2220, 802, 1371, 47)																
Negative	Ref				Ref				Ref				Ref			
Positive	1.556 (0.929-2.606)	0.093			1.101 (0.591-2.051)	0.762			2.127 (0.830-5.451)	0.116			/	/		
KI-67 (*n *= 2357, 799, 1377, 181)																
<5%	Ref				Ref				Ref				Ref			
5-25%	1.125 (0.424-2.984)	0.813			1.591 (0.158-16.018)	0.694			0.200 (0.017-2.386)	0.203			1.500 (0.353-6.376)	0.583		
>25%	1.084 (0.459-2.564)	0.854			3.405 (0.417-27.824)	0.253			0.548 (0.136-2.206)	0.398			2.109 (0.533-8.343)	0.287		
KNB Mutations (*n *= 853, 13, 836, 4)																
KRAS																
Mutation	Ref				Ref				Ref				Ref			
Negative	0.794 (0.574-1.097)	0.162			/	0.996			0.794 (0.571-1.104)	0.170			/	1.000		
NRAS																
Mutation	Ref				Ref				Ref				Ref			
Negative	1.660 (0.567-4.860)	0.355			/	/			1.610 (0.550-4.717)	0.385			/	/		
BRAF																
Mutation	Ref				Ref				Ref				Ref			
Negative	0.933 (0.394-2.209)	0.875			0.833 (0.041-16.995)	0.906			1.014 (0.405-2.539)	0.977			/	/		

## Discussion

This comprehensive study of 2370 patients across multiple gastrointestinal tumor types, including GC, CRC, and PC, reveals key insights into Trop2 expression patterns and their clinical significance. In our study, Trop2 is widely expressed across gastrointestinal malignancies, with particularly high prevalence in PC and GC. Its upregulation is strongly associated with aggressive tumor features (VI, PNI, advanced stage, low differentiation) and molecular alterations (pMMR status, MLH1-deficient, KRAS mutations, HER-2 positive, PD-L1 CPS negative, and high KI-67 expression). While corroborating established Trop2 expression patterns in gastrointestinal cancers, our study uniquely incorporates complete clinicopathological profiling and multivariate regression to delineate how tumor composition, staging, invasion status, and molecular characteristics collectively influence Trop2 expression. Trop2 expression exhibited distinct associations across gastrointestinal malignancies. At the pan-cancer level, Trop2 expression showed significant correlations with tumor type, presence of SRCC components, VI, and PNI. Notably, GC-specific analysis identified SRCC components and intestinal type Lauren classification as independent predictors of Trop2 expression. In CRC, VI as a significant factor correlated with Trop2 expression. Meanwhile, in pancreatic cancer, female sex and T1 stage were associated with Trop2 expression, revealing tumor-type specific regulatory patterns of Trop2 in gastrointestinal cancers. These findings suggest Trop2 as a potential biomarker for tumor aggressiveness and a promising therapeutic target, particularly in SRCC-rich, metastatic, or invasive cancers. This evidence provides a strong rationale for stratifying patient populations in future clinical investigations of Trop2-directed ADC therapies.

Previous studies have demonstrated that Trop2 promotes tumor progression, metastasis and therapy resistance through multiple signaling pathways including ERK/MAPK^15^, PI3K/AKT^16^, and Wnt/β-catenin.^17^ Characterized by its tumor-selective overexpression (50%-70% in GC, and 60%-80% in PC) with minimal expression in normal tissues, Trop2 has emerged as an ideal therapeutic target for ADC. Trop2-directed ADCs demonstrate several clinically relevant advantages that enhance their therapeutic potential. The antibody-mediated targeting mechanism ensures precise delivery of cytotoxic payloads while minimizing off-target effects, thereby improving the therapeutic index. Following target binding, these ADCs exhibit rapid and efficient internalization, facilitating intracellular release of potent payloads such as SN-38 or MMAE. Importantly, their capacity to exert bystander effects on adjacent tumor cells addresses a critical challenge in oncology by overcoming tumor heterogeneity, a major contributor to treatment resistance. However, the clinical translation of Trop2-targeted ADCs currently faces several significant challenges that require systematic investigation. A fundamental issue is establishing robust quantitative correlations between Trop2 expression levels and therapeutic response, necessitating the development of standardized companion diagnostic approaches, including refined IHC scoring systems and genomic assays. Concurrently, optimization of ADC structural components remains imperative, particularly with regard to linker stability, drug-to-antibody ratio optimization, and development of next-generation payloads with improved pharmacological properties.

Future research directions should prioritize three key areas to advance this therapeutic paradigm. First, implementation of biomarker-driven clinical trials incorporating comprehensive molecular profiling. Second, exploration of rational combination strategies, particularly with ICIs (e.g., PD-1/PD-L1 inhibitors) and molecularly targeted agents (e.g., Claudin18.2 inhibitors^26^), to maximize therapeutic synergy. Third, mechanistic studies elucidating resistance pathways, including potential defects in ADC internalization and activation of compensatory survival signaling networks. These collective efforts will facilitate the development of precision treatment algorithms for Trop2-expressing gastrointestinal malignancies, ultimately improving patient outcomes.

While this study enrolled 2370 digestive cancer patients and systematically characterized Trop2 expression patterns, several important limitations warrant emphasis. Firstly, as a single-center retrospective analysis from a tertiary hospital in China, potential selection bias may exist, particularly regarding underrepresented subgroups such as elderly patients or those with rare histological types. In addition, the exclusively Chinese cohort may limit the generalizability of Trop2 expression patterns to other ethnic or geographic populations, highlighting the need for validation in multicenter, multiethnic prospective studies. Secondly, although major digestive malignancies (GCs, CRCs, and PCs) were included, the analysis could not incorporate some less common subtypes (e.g., small bowel adenocarcinoma, gallbladder cancer, primary liver cancer, and appendiceal tumors) due to sample size constraints—a gap that may require addressing through establishing multicenter collaborative networks. It is also important to note that Trop2 testing at our institution is routinely performed only for pancreatic, colorectal, and GCs, while other tumor types such as esophageal cancer are not currently included in the routine testing panel. As a result, our cancer-type selection was partially constrained by clinical testing practices, introducing potential selection bias. Most notably, since Trop2 testing in digestive tumors was only implemented at our institution in 2022, comprehensive treatment response data and long-term survival outcomes remain unavailable. However, emerging clinical trials—including TROP2-targeted ADCs (e.g., NCT04152499 [MK-2870], NCT05060276), CAR-NK cell therapy (NCT06454890), and combination strategies with anti-PD-1 (NCT05941507)—have demonstrated preliminary efficacy in advanced solid tumors, supporting the therapeutic relevance of Trop2. To bridge this knowledge gap, we have initiated a structured follow-up program (quarterly assessments for ≥3 years) to collect efficacy end points, which will enable prognostic modeling correlating Trop2 expression with clinical outcomes.

## Conclusion

Trop2 is highly expressed in gastrointestinal malignancies, especially pancreatic and GCs, and correlates with aggressive features (VI, PNI, advanced stage, low differentiation) and molecular alterations (pMMR status, MLH1-deficient, KRAS mutations, HER-2 positive, PD-L1 CPS negative, and high KI-67 expression). These findings support Trop2 as both a prognostic biomarker and therapeutic target, particularly in SRCC-rich, metastatic, or invasive subtypes, justifying patient stratification for future Trop2-ADC clinical trials.

## Supplementary Material

oyaf320_Supplementary_Data

## Data Availability

All data in this study are included in the published article. All data generated in this study are available upon request from the corresponding author.
