# Supplementary material for: Trophoblast cell-surface antigen 2 expression in digestive neoplasms: a promising target for antibody-drug conjugates
Source: Oncologist. 2025 Oct 16;30(11):oyaf320. doi: 10.1093/oncolo/oyaf320 (PMC12659696; doi:10.1093/oncolo/oyaf320)
Supplement: oyaf320_Supplementary_Data [file oyaf320_supplementary_data.docx]

**Supplemental Table S1A.** Expression of Trop2 among different clinicopathological features: classification based on Trop2 expression status.

| **Characteristics** | **All cases (n=2370)** | | **Gastric cancer (n=804)** | | **Colorectal cancer (n=1384)** | | **Pancreatic cancer (n=182)** | |
| --- | --- | --- | --- | --- | --- | --- | --- | --- |
|  | **- (%)** | **+ (%)** | **- (%)** | **+ (%)** | **- (%)** | **+ (%)** | **- (%)** | **+ (%)** |
| Gender | n=540 | n=1830 | n=58 | n=746 | n=475 | n=909 | n=7 | n=175 |
| Male | 319 (59.07%) | 1173 (64.10%) | 29 (50.00%) | 532 (71.31%) | 284 (59.79%) | 534 (58.75%) | 6 (85.71%) | 107 (61.14%) |
| Female | 221 (40.93%) | 657 (35.90%) | 29 (50.00%) | 214 (28.69%) | 191 (40.21%) | 375 (41.25%) | 1 (14.29%) | 68 (38.86%) |
| Age (years) |  |  |  |  |  |  |  |  |
| <65 | 291 (53.89%) | 1055 (57.65%) | 29 (50.00%) | 408 (54.69%) | 259 (54.53%) | 538 (59.19%) | 3 (42.86%) | 109 (62.29%) |
| ≥65 | 249 (46.11%) | 775 (42.35%) | 29 (50.00%) | 338 (45.31%) | 216 (45.47%) | 371 (40.81%) | 4 (57.14%) | 66 (37.71%) |
| Tumor sites |  |  |  |  |  |  |  |  |
| Stomach | 58 (10.74%) | 746 (40.77%) | 58 (100.00%) | 746 (100.00%) |  |  |  |  |
| Gastroesophageal junction |  |  | 1 (1.72%) | 18 (2.41%) |  |  |  |  |
| Cardia |  |  | 7 (12.07%) | 140 (18.77%) |  |  |  |  |
| Gastric body |  |  | 13 (22.41%) | 174 (23.32%) |  |  |  |  |
| Gastric fundus |  |  | 1 (1.72%) | 23 (3.08%) |  |  |  |  |
| Gastric antrum |  |  | 24 (41.38%) | 267 (35.79%) |  |  |  |  |
| Lesser curvature |  |  | 9 (15.52%) | 99 (13.27%) |  |  |  |  |
| Pylorus |  |  | 2 (3.45%) | 6 (0.80%) |  |  |  |  |
| Gastric remnant |  |  | 1 (1.72%) | 19 (2.55%) |  |  |  |  |
| Colorectum | 475 (87.96%) | 909 (49.67%) |  |  | 475 (100.00%) | 909 (100.00%) |  |  |
| Colon |  |  |  |  | 179 (37.68%) | 319 (35.09%) |  |  |
| Sigmoid colon |  |  |  |  | 91 (19.16%) | 137 (15.07%) |  |  |
| Rectum |  |  |  |  | 202 (42.53%) | 446 (49.06%) |  |  |
| Cecum |  |  |  |  | 3 (0.63%) | 7 (0.77%) |  |  |
| Pancreas | 7 (1.30%) | 175 (9.56%) |  |  |  |  | 7 (100.00%) | 175 (100.00%) |
| Histological classification |  |  |  |  |  |  |  |  |
| Adenocarcinoma | 533 (98.70%) | 1811 (98.96%) | 57 (98.28%) | 732 (98.12%) | 471 (99.16%) | 907 (99.78%) | 5 (71.43%) | 172 (98.29%) |
| Others | 7 (1.30%) | 19 (1.04%) | 1 (1.72%) | 14 (1.88%) | 4 (0.84%) | 2 (0.22%) | 2 (28.57%) | 3 (1.71%) |
| Signet ring cell carcinoma components (SRCC) |  |  |  |  |  |  |  |  |
| Yes | 15 (2.78%) | 168 (9.18%) | 10 (17.24%) | 148 (19.84%) | 5 (1.05%) | 18 (1.98%) | 0 (0.00%) | 2 (1.14%) |
| No | 525 (97.22%) | 1662 (90.82%) | 48 (82.76%) | 598 (80.16%) | 470 (98.95%) | 891 (98.02%) | 7 (100.00%) | 173 (98.86%) |
| Mucinous adenocarcinoma components (MAC) |  |  |  |  |  |  |  |  |
| Yes | 51 (9.44%) | 165 (9.02%) | 1 (1.72%) | 33 (4.42%) | 50 (10.53%) | 126 (13.86%) | 0 (0.00%) | 6 (3.43%) |
| No | 489 (90.56%) | 1665 (90.98%) | 57 (98.28%) | 713 (95.58%) | 425 (89.47%) | 783 (86.14%) | 7 (100.00%) | 169 (96.57%) |
| Lauren classification (n=671, 671, 0, 0) | n=48 | n=623 | n=48 | n=623 | / | / | / | / |
| Intestinal type | 14 (29.17%) | 157 (25.20%) | 14 (29.17%) | 157 (25.20%) |  |  |  |  |
| Diffuse type | 18 (37.50%) | 151 (24.24%) | 18 (37.50%) | 151 (24.24%) |  |  |  |  |
| Mixed type | 15 (31.25%) | 284 (45.59%) | 15 (31.25%) | 284 (45.59%) |  |  |  |  |
| Indeterminate type | 1 (2.08%) | 31 (4.98%) | 1 (2.08%) | 31 (4.98%) |  |  |  |  |
| Differentiation degree |  |  |  |  |  |  |  |  |
| Poor | 220 (40.74%) | 1093 (59.73%) | 44 (75.86%) | 595 (79.76%) | 170 (35.79%) | 387 (42.57%) | 6 (85.71%) | 111 (63.43%) |
| Moderate | 268 (49.63%) | 582 (31.80%) | 10 (10.86%) | 81 (10.86%) | 257 (54.11%) | 457 (50.28%) | 1 (14.29%) | 44 (25.14%) |
| Well | 52 (9.63%) | 155 (8.47%) | 4 (9.38%) | 70 (9.38%) | 48 (10.11%) | 65 (7.15%) | 0 (0.00%) | 20 (11.43%) |
| T stage |  |  |  |  |  |  |  |  |
| 1 | 42 (7.78%) | 245 (13.39%) | 17 (29.31%) | 172 (23.06%) | 25 (5.26%) | 46 (5.06%) | 0 (0.00%) | 27 (15.43%) |
| 2 | 79 (14.63%) | 276 (15.08%) | 8 (13.79%) | 82 (10.99%) | 69 (14.53%) | 101 (11.11%) | 2 (28.57%) | 93 (53.14%) |
| 3 | 316 (58.52%) | 824 (45.03%) | 19 (32.76%) | 250 (33.51%) | 295 (62.11%) | 547 (60.18%) | 2 (28.57%) | 27 (15.43%) |
| 4 | 68 (12.59%) | 330 (18.03%) | 10 (17.24%) | 180 (24.13%) | 56 (11.79%) | 134 (14.74%) | 2 (28.57%) | 16 (9.14%) |
| NA | 35 (6.48%) | 155 (8.47%) | 4 (6.90%) | 62 (8.31%) | 30 (6.32%) | 81 (8.91%) | 1 (14.29%) | 12 (6.86%) |
| N stage |  |  |  |  |  |  |  |  |
| 0 | 299 (55.37%) | 780 (42.62%) | 29 (50.00%) | 308 (41.29%) | 265 (55.79%) | 381 (41.91%) | 5 (71.43%) | 91 (52.00%) |
| 1 | 128 (23.70%) | 420 (22.95%) | 9 (15.52%) | 110 (14.75%) | 119 (25.05%) | 252 (27.72%) | 0 (0.00%) | 58 (33.14%) |
| 2 | 66 (12.22%) | 323 (17.65%) | 4 (6.90%) | 114 (15.28%) | 61 (12.84%) | 195 (21.45%) | 1 (14.29%) | 14 (8.00%) |
| 3 | 12 (2.22%) | 152 (8.31%) | 12 (20.69%) | 152 (20.38%) | / | / | / | / |
| NA | 35 (6.48%) | 155 (8.47%) | 4 (6.90%) | 62 (8.31%) | 30 (6.32%) | 81 (8.91%) | 1 (14.29%) | 12 (6.86%) |
| M stage |  |  |  |  |  |  |  |  |
| 0 | 465 (86.11%) | 1523 (83.22%) | 53 (91.38%) | 645 (86.46%) | 407 (85.68%) | 746 (82.07%) | 5 (71.43%) | 132 (75.43%) |
| 1 | 62 (11.48%) | 245 (13.39%) | 4 (6.90%) | 74 (9.92%) | 56 (11.79%) | 132 (14.52%) | 2 (28.57%) | 39 (22.29%) |
| NA | 13 (2.41%) | 62 (3.39%) | 1 (1.72%) | 27 (3.62%) | 12 (2.53%) | 31 (3.41%) | 0 (0.00%) | 4 (2.29%) |
| Stage |  |  |  |  |  |  |  |  |
| I | 102 (18.89%) | 382 (20.87%) | 21 (36.21%) | 208 (27.88%) | 79 (16.63%) | 110 (12.10%) | 2 (28.57%) | 64 (36.57%) |
| II | 190 (35.19%) | 456 (24.92%) | 15 (25.86%) | 155 (20.78%) | 175 (36.84%) | 258 (28.38%) | 0 (0.00%) | 43 (24.57%) |
| III | 173 (32.04%) | 685 (37.43%) | 17 (29.31%) | 282 (37.80%) | 153 (32.21%) | 378 (41.58%) | 3 (42.86%) | 25 (14.29%) |
| IV | 62 (11.48%) | 245 (13.39%) | 4 (6.90%) | 74 (9.92%) | 56 (11.79%) | 132 (14.52%) | 2 (28.57%) | 39 (22.29%) |
| NA | 13 (2.41%) | 62 (3.39%) | 1 (1.72%) | 27 (3.62%) | 12 (88.21%) | 31 (3.41%) | 0 (0.00%) | 4 (2.29%) |
| Metastasis |  |  |  |  |  |  |  |  |
| Yes | 62 (11.48%) | 245 (13.39%) | 4 (6.90%) | 74 (9.92%) | 56 (11.79%) | 132 (3.41%) | 2 (28.57%) | 39 (22.29%) |
| No | 478 (88.52%) | 1585 (86.61%) | 54 (93.10%) | 672 (90.08%) | 419 (88.21%) | 777 (85.48%) | 5 (71.43%) | 136 (77.71%) |
| Vascular invasion (VI, n=2109, 723, 1256, 130) | n=492 | n=1617 | n=53 | n=670 | n=434 | n=822 | n=5 | n=125 |
| Yes | 132 (26.83%) | 656 (40.57%) | 17 (32.08%) | 323 (48.21%) | 112 (25.81%) | 295 (35.89%) | 3 (60.00%) | 38 (30.40%) |
| No | 360 (73.17%) | 961 (59.43%) | 36 (67.92%) | 347 (51.79%) | 322 (74.19%) | 527 (64.11%) | 2 (40.00%) | 87 (69.60%) |
| Perineural invasion (PNI, n=2109, 723, 1256, 130) | n=492 | n=1617 | n=53 | n=670 | n=434 | n=822 | n=5 | n=125 |
| Yes | 184 (37.40%) | 878 (54.30%) | 25 (47.17%) | 361 (53.88%) | 158 (36.41%) | 406 (49.39%) | 1 (20.00%) | 111 (88.80%) |
| No | 308 (62.60%) | 739 (45.70%) | 28 (52.83%) | 309 (46.12%) | 276 (63.59%) | 416 (50.61%) | 4 (80.00%) | 14 (11.20%) |
| MMR status |  |  |  |  |  |  |  |  |
| pMMR | 496 (91.85%) | 1751 (95.68%) | 49 (84.48%) | 716 (95.98%) | 441 (92.84%) | 860 (94.61%) | 6 (85.71%) | 175 (100.00%) |
| dMMR | 44 (8.15%) | 79 (4.32%) | 9 (15.52%) | 30 (4.02%) | 34 (7.16%) | 49 (5.39%) | 1 (14.29%) | 0 (0.00%) |
| Functional defects of tumor mismatch repair genes (n=123, 39, 83, 1) | n=44 | n=79 | n=9 | n=30 | n=34 | n=49 | n=1 | n=0 |
| MSH2 | 12 (27.27%) | 11 (13.92%) | 0 (0.00%) | 0 (0.00%) | 12 (35.29%) | 11 (22.45%) | 0 (0.00%) | / |
| MLH1 | 25 (56.82%) | 58 (73.42%) | 7 (77.78%) | 28 (93.33%) | 18 (52.94%) | 30 (61.22%) | 0 (0.00%) | / |
| MSH6 | 14 (31.82%) | 13 (16.46%) | 0 (0.00%) | 0 (0.00%) | 13 (38.24%) | 13 (26.53%) | 1 (100.00%) | / |
| PMS2 | 31 (70.45%) | 63 (79.75%) | 9 (100.00%) | 29 (96.67%) | 22 (64.71%) | 34 (69.39%) | 0 (0.00%) | / |
| PD-L1 CPS (n=34, 27, 5, 2) | n=0 | n=34 | n=58 | n=27 | n=0 | n=5 | n=0 | n=2 |
| Negative | / | 13 (38.24%) | 0 (0.00%) | 9 (33.33%) | / | 3 (60.00%) | / | 1 (50.00%) |
| Positive | / | 21 (61.76%) | 58 (100.00%) | 18 (66.67%) | / | 2 (40.00%) | / | 1 (50.00%) |
| HER-2 (n=2220, 802, 1371, 47) | n=531 | n=1689 | n=58 | n=744 | n=470 | n=901 | n=3 | n=44 |
| Negative | 525 (98.87%) | 1629 (96.45%) | 55 (94.83%) | 700 (94.09%) | 467 (99.36%) | 885 (98.22%) | 3 (100.00%) | 44 (100.00%) |
| Positive | 6 (1.13%) | 60 (3.55%) | 3 (5.17%) | 44 (5.91%) | 3 (0.64%) | 16 (1.78%) | 0 (0.00%) | 0 (0.00%) |
| KI-67 (n=2357, 799, 1377, 181) | n=537 | n=1820 | n=57 | n=742 | n=473 | n=904 | n=7 | n=174 |
| <5% | 7 (1.30%) | 21 (1.15%) | 2 (3.51%) | 6 (0.81%) | 3 (0.63%) | 6 (0.66%) | 2 (28.57%) | 9 (5.17%) |
| 5-25% | 9 (1.68%) | 79 (4.34%) | 5 (8.77%) | 22 (2.96%) | 4 (0.85%) | 7 (0.77%) | 0 (0.00%) | 50 (28.74%) |
| >25% | 521 (97.02%) | 1720 (94.51%) | 50 (87.72%) | 714 (96.23%) | 466 (98.52%) | 891 (98.56%) | 5 (71.43%) | 115 (66.09%) |
| KNB Mutations (n=853, 13, 836, 4) |  |  |  |  |  |  |  |  |
| KRAS | n=291 | n=562 | n=0 | n=13 | n=290 | n=546 | n=1 | n=3 |
| Mutation | 112 (38.49%) | 292 (51.96%) | / | 1 (7.69%) | 112 (38.62%) | 289 (52.93%) | 0 (0.00%) | 2 (66.67%) |
| Negative | 179 (61.51%) | 270 (48.04%) | / | 12 (92.31%) | 178 (61.38%) | 257 (47.07%) | 1 (100.00%) | 1 (33.33%) |
| NRAS |  |  |  |  |  |  |  |  |
| Mutation | 9 (3.09%) | 18 (3.20%) | / | 0 (0.00%) | 9 (3.10%) | 18 (3.30%) | 0 (0.00%) | 0 (0.00%) |
| Negative | 282 (96.91%) | 544 (96.80%) | / | 13 (100.00%) | 281 (96.90%) | 528 (96.70%) | 1 (100.00%) | 3 (100.00%) |
| BRAF |  |  |  |  |  |  |  |  |
| Mutation | 8 (2.75%) | 22 (3.91%) | / | 2 (15.38%) | 8 (2.76%) | 20 (3.66%) | 0 (0.00%) | 0 (0.00%) |
| Negative | 283 (97.25%) | 540 (96.09%) | / | 11 (84.62%) | 282 (97.24%) | 526 (96.34%) | 1 (100.00%) | 3 (100.00%) |

**Supplemental Table S1B.** Expression of Trop2 among different clinicopathological features: classification based on Trop2 regional area.

| **Characteristics** | **All cases (n=2370)** | | | | **Gastric cancer (n=804)** | | | | **Colorectal cancer (n=1384)** | | | | **Pancreatic cancer (n=182)** | | | |
| --- | --- | --- | --- | --- | --- | --- | --- | --- | --- | --- | --- | --- | --- | --- | --- | --- |
|  | **<25% (%)** | **25-50% (%)** | **50-75% (%)** | **>75% (%)** | **<25% (%)** | **25-50% (%)** | **50-75% (%)** | **>75% (%)** | **<25% (%)** | **25-50% (%)** | **50-75% (%)** | **>75% (%)** | **<25% (%)** | **25-50% (%)** | **50-75% (%)** | **>75% (%)** |
| Gender | n=602 | n=603 | n=232 | n=393 | n=166 | n=282 | n=113 | n=185 | n=432 | n=265 | n=95 | n=117 | n=4 | n=56 | n=24 | n=91 |
| Male | 369 (61.30%) | 395 (65.51%) | 146 (62.93%) | 263 (66.92%) | 117 (70.48%) | 201 (71.28%) | 80 (70.80%) | 134 (72.43%) | 248 (57.41%) | 158 (59.62%) | 55 (57.89%) | 73 (62.39%) | 4 (100.00%) | 36 (64.29%) | 11 (45.83%) | 56 (61.54%) |
| Female | 233 (38.70%) | 208 (34.49%) | 86 (37.07%) | 130 (33.08%) | 49 (29.52%) | 81 (28.72%) | 33 (29.20%) | 51 (27.57%) | 184 (42.59%) | 107 (40.38%) | 40 (42.11%) | 44 (37.61%) | 0 (0.00%) | 20 (35.71%) | 13 (54.17%) | 35 (38.46%) |
| Age (years) |  |  |  |  |  |  |  |  |  |  |  |  |  |  |  |  |
| <65 | 345 (57.31%) | 339 (56.22%) | 151 (65.09%) | 220 (55.98%) | 96 (57.83%) | 163 (57.80%) | 69 (61.06%) | 80 (43.24%) | 245 (56.71%) | 146 (55.09%) | 68 (71.58%) | 79 (67.52%) | 4 (100.00%) | 30 (53.57%) | 14 (58.33%) | 61 (67.03%) |
| ≥65 | 257 (42.69%) | 264 (43.78%) | 81 (34.91%) | 173 (44.02%) | 70 (42.17%) | 119 (42.20%) | 44 (38.94%) | 105 (56.76%) | 187 (43.29%) | 119 (44.91%) | 27 (28.42%) | 38 (32.48%) | 0 (0.00%) | 26 (46.43%) | 10 (41.67%) | 30 (32.97%) |
| Tumor sites |  |  |  |  |  |  |  |  |  |  |  |  |  |  |  |  |
| Stomach | 166 (27.57%) | 282 (46.77%) | 113 (48.71%) | 185 (47.07%) | 166 (100.00%) | 282 (100.00%) | 113 (100.00%) | 185 (100.00%) | / | / | / | / | / | / | / | / |
| Gastroesophageal junction |  |  |  |  | 1 (0.60%) | 9 (3.19%) | 4 (3.54%) | 4 (2.17%) |  |  |  |  |  |  |  |  |
| Cardia |  |  |  |  | 31 (18.67%) | 40 (14.18%) | 31 (27.43%) | 38 (20.65%) |  |  |  |  |  |  |  |  |
| Gastric body |  |  |  |  | 39 (23.49%) | 69 (24.47%) | 26 (23.01%) | 40 (21.74%) |  |  |  |  |  |  |  |  |
| Gastric fundus |  |  |  |  | 6 (3.61%) | 9 (3.19%) | 3 (2.65%) | 5 (2.72%) |  |  |  |  |  |  |  |  |
| Gastric antrum |  |  |  |  | 65 (39.16%) | 105 (37.23%) | 32 (28.32%) | 65 (35.33%) |  |  |  |  |  |  |  |  |
| Lesser curvature |  |  |  |  | 18 (10.84%) | 42 (14.89%) | 15 (13.27%) | 24 (13.04%) |  |  |  |  |  |  |  |  |
| Pylorus |  |  |  |  | 3 (1.81%) | 1 (0.35%) | 0 (0.00%) | 2 (1.09%) |  |  |  |  |  |  |  |  |
| Gastric remnant |  |  |  |  | 3 (1.81%) | 7 (2.48%) | 2 (1.77%) | 7 (3.80%) |  |  |  |  |  |  |  |  |
| Colorectum | 432 (71.76%) | 265 (43.95%) | 95 (40.95%) | 117 (29.77%) | / | / | / | / | 432 (100.00%) | 265 (100.00%) | 95 (100.00%) | 117 (100.00%) | / | / | / | / |
| Colon |  |  |  |  |  |  |  |  | 135 (31.25%) | 102 (38.49%) | 43 (45.26%) | 39 (33.33%) |  |  |  |  |
| Sigmoid colon |  |  |  |  |  |  |  |  | 66 (15.28%) | 42 (15.85%) | 10 (10.53%) | 19 (16.24%) |  |  |  |  |
| Rectum |  |  |  |  |  |  |  |  | 229 (53.01%) | 117 (44.15%) | 42 (44.21%) | 58 (49.57%) |  |  |  |  |
| Cecum |  |  |  |  |  |  |  |  | 2 (0.46%) | 4 (1.51%) | 0 (0.00%) | 1 (0.85%) |  |  |  |  |
| Pancreas | 4 (0.66%) | 56 (9.29%) | 24 (10.34%) | 91 (23.16%) | / | / | / | / | / | / | / | / | 4 (100.00%) | 56 (100.00%) | 24 (100.00%) | 91 (100.00%) |
| Histological classification |  |  |  |  |  |  |  |  |  |  |  |  |  |  |  |  |
| Adenocarcinoma | 594 (98.67%) | 601 (99.67%) | 228 (98.28%) | 388 (98.73%) | 160 (96.39%) | 280 (99.29%) |  |  |  |  |  |  |  |  |  |  |
| Others | 8 (1.33%) | 2 (0.33%) | 4 (1.72%) | 5 (1.27%) | 6 (3.61%) | 2 (0.71%) |  |  |  |  |  |  |  |  |  |  |
| Signet ring cell carcinoma components (SRCC) |  |  |  |  |  |  |  |  |  |  |  |  |  |  |  |  |
| Yes | 36 (5.98%) | 64 (10.61%) | 23 (9.91%) | 45 (11.45%) | 33 (19.88%) | 56 (19.86%) |  |  |  |  |  |  |  |  |  |  |
| No | 566 (94.02%) | 539 (89.39%) | 209 (90.09%) | 348 (88.55%) | 133 (80.12%) | 226 (80.14%) |  |  |  |  |  |  |  |  |  |  |
| Mucinous adenocarcinoma components (MAC) |  |  |  |  |  |  |  |  |  |  |  |  |  |  |  |  |
| Yes | 53 (8.80%) | 56 (9.29%) | 27 (11.64%) | 29 (7.38%) | 6 (3.61%) | 13 (4.61%) |  |  |  |  |  |  |  |  |  |  |
| No | 549 (91.20%) | 547 (90.71%) | 205 (88.36%) | 364 (92.62%) | 160 (96.39%) | 269 (95.39%) |  |  |  |  |  |  |  |  |  |  |
| Lauren classification (n=671, 671, 0, 0) | n=144 | n=234 | n=95 | n=150 | n=144 | n=234 | n=95 | n=150 | / | / | / | / | / | / | / | / |
| Intestinal type | 38 (26.39%) | 48 (20.51%) | 26 (27.37%) | 45 (30.00%) | 38 (26.39%) | 48 (20.51%) | 26 (27.37%) | 45 (30.00%) |  |  |  |  |  |  |  |  |
| Diffuse type | 35 (24.31%) | 72 (30.77%) | 17 (17.89%) | 27 (18.00%) | 35 (24.31%) | 72 (30.77%) | 17 (17.89%) | 27 (18.00%) |  |  |  |  |  |  |  |  |
| Mixed type | 63 (43.75%) | 104 (44.44%) | 46 (48.42%) | 71 (47.33%) | 63 (43.75%) | 104 (44.44%) | 46 (48.42%) | 71 (47.33%) |  |  |  |  |  |  |  |  |
| Indeterminate type | 8 (5.56%) | 10 (4.27%) | 6 (6.32%) | 7 (4.67%) | 8 (5.56%) | 10 (4.27%) | 6 (6.32%) | 7 (4.67%) |  |  |  |  |  |  |  |  |
| Differentiation degree |  |  |  |  |  |  |  |  |  |  |  |  |  |  |  |  |
| Poor | 278 (46.18%) | 386 (64.01%) | 163 (70.26%) | 266 (67.68%) | 131 (78.92%) | 234 (82.98%) | 90 (79.65%) | 140 (75.68%) | 143 (33.10%) | 123 (46.42%) | 54 (56.84%) | 67 (57.26%) | 4 (100.00%) | 29 (51.79%) | 19 (79.17%) | 59 (64.84%) |
| Moderate | 275 (45.68%) | 168 (27.86%) | 56 (24.14%) | 83 (21.12%) | 18 (10.84%) | 23 (8.16%) | 15 (13.27%) | 25 (13.51%) | 257 (.59%49) | 126 (47.55%) | 38 (40.00%) | 36 (30.77%) | 0 (0.00%) | 19 (33.93%) | 3 (12.50%) | 22 (24.18%) |
| Well | 49 (8.13%) | 49 (8.13%) | 13 (5.60%) | 44 (11.20%) | 17 (10.24%) | 25 (8.87%) | 8 (.7%08) | 20 (10.81%) | 32 (7.41%) | 16 (6.04%) | 3 (3.16%) | 14 (11.97%) | 0 (0.00%) | 8 (14.29%) | 2 (8.33%) | 10 (10.99%) |
| T stage |  |  |  |  |  |  |  |  |  |  |  |  |  |  |  |  |
| 1 | 69 (11.46%) | 95 (15.75%) | 27 (11.64%) | 54 (13.74%) | 41 (24.70%) | 74 (26.24%) | 21 (18.58%) | 36 (19.46%) | 27 (6.25%) | 12 (4.53%) | 4 (4.21%) | 3 (2.56%) | 1 (25.00%) | 9 (16.07%) | 2 (8.33%) | 15 (16.48%) |
| 2 | 83 (13.79%) | 75 (12.44%) | 35 (15.09%) | 83 (21.12%) | 20 (12.05%) | 24 (8.51%) | 16 (14.16%) | 22 (11.89%) | 60 (13.89%) | 24 (9.06%) | 5 (5.26%) | 12 (10.26%) | 3 (75.00%) | 27 (48.21%) | 14 (58.33%) | 49 (53.85%) |
| 3 | 327 (54.32%) | 260 (43.12%) | 107 (46.12%) | 130 (33.08%) | 54 (32.53%) | 101 (35.82%) | 44 (38.94%) | 51 (27.57%) | 273 (63.19%) | 153 (57.74%) | 57 (60.00%) | 64 (54.70%) | 0 (0.00%) | 6 (10.71%) | 6 (25.00%) | 15 (16.48%) |
| 4 | 76 (12.62%) | 127 (21.06%) | 49 (21.12%) | 78 (19.85%) | 39 (23.49%) | 66 (23.40%) | 25 (22.12%) | 50 (27.03%) | 37 (8.56%) | 52 (19.62%) | 24 (25.26%) | 21 (17.95%) | 0 (0.00%) | 9 (16.07%) | 0 (0.00%) | 7 (7.69%) |
| NA | 47 (7.81%) | 46 (7.63%) | 14 (6.03%) | 48 (12.21%) | 12 (7.23%) | 17 (6.03%) | 7 (6.19%) | 26 (14.05%) | 35 (8.10%) | 24 (9.06%) | 5 (5.26%) | 17 (14.53%) | 0 (0.00%) | 5 (8.93%) | 2 (8.33%) | 5 (5.49%) |
| N stage |  |  |  |  |  |  |  |  |  |  |  |  |  |  |  |  |
| 0 | 292 (48.50%) | 252 (41.79%) | 88 (37.93%) | 148 (37.66%) | 73 (43.98%) | 123 (43.62%) | 41 (36.28%) | 71 (38.38%) | 217 (50.23%) | 98 (36.98%) | 35 (36.84%) | 31 (26.50%) | 2 (50.00%) | 31 (55.36%) | 12 (50.00%) | 46 (50.55%) |
| 1 | 146 (24.25%) | 128 (21.23%) | 59 (25.43%) | 87 (22.14%) | 22 (13.25%) | 44 (15.6%) | 23 (20.35%) | 21 (11.35%) | 122 (28.24%) | 69 (26.04%) | 27 (28.42%) | 34 (29.06%) | 2 (50.00%) | 15 (26.79%) | 9 (37.5%) | 32 (35.16%) |
| 2 | 82 (13.62%) | 121 (20.07%) | 46 (19.83%) | 74 (18.83%) | 24 (14.46%) | 42 (14.89%) | 17 (15.04%) | 31 (16.76%) | 58 (13.43%) | 74 (27.92%) | 28 (29.47%) | 35 (29.91%) | 0 (0.00%) | 5 (8.93%) | 1 (4.17%) | 8 (8.79%) |
| 3 | 35 (5.81%) | 56 (9.29%) | 15 (6.47%) | 46 (11.7%) | 35 (21.08%) | 56 (19.86%) | 25 (22.12%) | 36 (19.46%) | / | / | / | / | / | / | / | / |
| NA | 47 (7.81%) | 46 (7.63%) | 14 (6.03%) | 48 (12.21%) | 12 (7.23%) | 17 (6.03%) | 7 (6.19%) | 26 (14.05%) | 35 (8.10%) | 24 (9.06%) | 5 (5.26%) | 17 (14.53%) | 0 (0.00%) | 5 (8.93%) | 2 (8.33%) | 5 (5.49%) |
| M stage |  |  |  |  |  |  |  |  |  |  |  |  |  |  |  |  |
| 0 | 511 (84.88%) | 509 (84.41%) | 199 (85.78%) | 304 (77.35%) | 144 (86.75%) | 253 (89.72%) | 98 (86.73%) | 150 (81.08%) | 364 (84.26%) | 213 (80.38%) | 82 (86.32%) | 87 (74.36%) | 3 (75.00%) | 43 (76.79%) | 19 (79.17%) | 67 (73.63%) |
| 1 | 64 (10.63%) | 75 (12.44%) | 29 (12.50%) | 77 (19.59%) | 12 (7.23%) | 22 (7.80%) | 12 (10.62%) | 28 (15.14%) | 51 (11.81%) | 42 (15.85%) | 13 (13.68%) | 26 (22.22%) | 1 (25.00%) | 11 (19.64%) | 4 (16.67%) | 23 (25.27%) |
| NA | 27 (4.49%) | 19 (3.15%) | 4 (1.72%) | 12 (3.05%) | 10 (6.02%) | 7 (2.48%) | 3 (2.65%) | 7 (3.78%) | 17 (3.94%) | 10 (3.77%) | 0 (0.00%) | 4 (3.42%) | 0 (0.00%) | 2 (3.57%) | 1 (4.17%) | 1 (1.10%) |
| Stage |  |  |  |  |  |  |  |  |  |  |  |  |  |  |  |  |
| I | 121 (20.10%) | 130 (21.56%) | 42 (18.10%) | 89 (22.65%) | 52 (31.33%) | 81 (28.72%) | 29 (25.66%) | 46 (24.86%) | 67 (15.51%) | 29 (10.94%) | 4 (4.21%) | 10 (8.55%) | 2 (50.00%) | 20 (35.71%) | 9 (37.50%) | 33 (36.26%) |
| II | 173 (28.74%) | 142 (23.55%) | 65 (28.02%) | 76 (19.34%) | 31 (18.67%) | 66 (23.40%) | 25 (22.12%) | 33 (17.84%) | 141 (32.64%) | 66 (24.91%) | 31 (32.63%) | 20 (17.09%) | 1 (25.00%) | 10 (17.86%) | 9 (37.50%) | 23 (25.27%) |
| III | 217 (36.05%) | 237 (39.30%) | 92 (39.66%) | 139 (35.37%) | 61 (36.75%) | 106 (37.59%) | 44 (38.94%) | 71 (38.38%) | 156 (36.11%) | 118 (44.53%) | 47 (49.47%) | 57 (48.72%) | 0 (0.00%) | 13 (23.21%) | 1 (4.17%) | 11 (12.09%) |
| IV | 64 (10.63%) | 75 (12.44%) | 29 (12.50%) | 77 (19.59%) | 12 (7.23%) | 22 (7.80%) | 12 (10.62%) | 28 (15.14%) | 51 (11.81%) | 42 (15.85%) | 13 (13.68%) | 26 (22.22%) | 1 (25.00%) | 11 (19.64%) | 4 (16.67%) | 23 (25.27%) |
| NA | 27 (89.37%) | 19 (87.56%) | 4 (1.72%) | 12 (3.05%) | 10 (6.02%) | 7 (2.48%) | 3 (2.65%) | 7 (3.78%) | 17 (3.94%) | 10 (3.77%) | 0 (0.00%) | 4 (3.42%) | 0 (0.00%) | 2 (3.57%) | 1 (4.17%) | 1 (1.10%) |
| Metastasis |  |  |  |  |  |  |  |  |  |  |  |  |  |  |  |  |
| Yes | 64 (10.63%) | 75 (29.33%) | 29 (12.50%) | 77 (19.59%) | 12 (7.23%) | 22 (7.80%) | 12 (10.62%) | 28 (15.14%) | 51 (11.81%) | 42 (15.85%) | 13 (13.68%) | 26 (22.22%) | 1 (25.00%) | 11 (19.64%) | 4 (16.67%) | 23 (25.27%) |
| No | 538 (89.37%) | 528 (70.67%) | 203 (87.50%) | 316 (80.41%) | 154 (92.77%) | 260 (92.20%) | 101 (89.38%) | 157 (84.86%) | 381 (88.19%) | 223 (84.15%) | 82 (86.32%) | 91 (77.78%) | 3 (75.00%) | 45 (80.36%) | 20 (83.33%) | 68 (74.73%) |
| Vascular invasion (VI, n=2109, 723, 1256, 130) | n=556 | n=533 | n=208 | n=320 | n=153 | n=258 | n=101 | n=158 | n=400 | n=236 | n=89 | n=97 | n=3 | n=39 | n=18 | n=65 |
| Yes | 178 (32.01%) | 219 (41.09%) | 99 (47.60%) | 160 (50.00%) | 65 (42.48%) | 122 (47.29%) | 52 (51.49%) | 84 (53.16%) | 113 (28.25%) | 90 (38.14%) | 41 (46.07%) | 51 (52.58%) | 0 (0.00%) | 7 (17.95%) | 6 (33.33%) | 25 (38.46%) |
| No | 378 (67.99%) | 314 (58.91%) | 109 (52.40%) | 160 (50.00%) | 88 (57.52%) | 136 (52.71%) | 49 (48.51%) | 74 (46.84%) | 287 (71.75%) | 146 (61.86%) | 48 (53.93%) | 46 (47.42%) | 3 (100.00%) | 32 (82.05%) | 12 (66.67%) | 40 (61.54%) |
| Perineural invasion (PNI, n=2109, 723, 1256, 130) | n=556 | n=533 | n=208 | n=320 | n=153 | n=258 | n=101 | n=158 | n=400 | n=236 | n=89 | n=97 | n=3 | n=39 | n=18 | n=65 |
| Yes | 267 (48.02%) | 287 (53.85%) | 115 (55.29%) | 209 (65.31%) | 79 (51.63%) | 138 (53.49%) | 51 (50.50%) | 93 (58.86%) | 186 (46.50%) | 117 (49.58%) | 48 (53.93%) | 55 (56.70%) | 2 (66.67%) | 32 (82.05%) | 16 (88.89%) | 61 (93.85%) |
| No | 289 (51.98%) | 246 (46.15%) | 93 (44.71%) | 111 (34.69%) | 74 (48.37%) | 120 (46.51%) | 50 (49.50%) | 65 (41.14%) | 214 (53.50%) | 119 (50.42%) | 41 (46.07%) | 42 (43.30%) | 1 (33.33%) | 7 (17.95%) | 2 (11.11%) | 4 (6.15%) |
| MMR status |  |  |  |  |  |  |  |  |  |  |  |  |  |  |  |  |
| pMMR | 573 (95.18%) | 578 (95.85%) | 216 (93.10%) | 384 (97.71%) | 159 (95.78%) | 271 (96.10%) | 105 (92.92%) | 181 (97.84%) | 410 (94.91%) | 251 (94.72%) | 87 (91.58%) | 112 (95.73%) | 4 (100.00%) | 56 (100.00%) | 24 (100.00%) | 91 (100.00%) |
| dMMR | 29 (4.82%) | 25 (4.15%) | 16 (6.90%) | 9 (2.29%) | 7 (4.22%) | 11 (3.90%) | 8 (7.08%) | 4 (2.16%) | 22 (5.09%) | 14 (5.28%) | 8 (8.42%) | 5 (4.27%) | 0 (0.00%) | 0 (0.00%) | 0 (0.00%) | 0 (0.00%) |
| Functional defects of tumor mismatch repair genes (n=123, 39, 83, 1) | n=29 | n=25 | n=16 | n=9 | n=7 | n=11 | n=8 | n=4 | n=22 | n=14 | n=8 | n=5 | / | / | / | / |
| MSH2 | 4 (13.79%) | 4 (16.00%) | 1 (6.25%) | 2 (22.22%) | 0 (0.00%) | 0 (0.00%) | 0 (0.00%) | 0 (0.00%) | 4 (18.18%) | 4 (28.57%) | 1 (12.50%) | 2 (40.00%) |  |  |  |  |
| MLH1 | 18 (62.07%) | 19 (76.00%) | 14 (87.50%) | 7 (77.78%) | 6 (85.71%) | 11 (100.00%) | 7 (87.50%) | 4 (100.00%) | 12 (54.55%) | 8 (57.14%) | 7 (87.50%) | 3 (60.00%) |  |  |  |  |
| MSH6 | 7 (24.14%) | 4 (16.00%) | 1 (6.25%) | 1 (11.11%) | 0 (0.00%) | 0 (0.00%) | 0 (0.00%) | 0 (0.00%) | 7 (31.82%) | 4 (28.57%) | 1 (12.50%) | 1 (20.00%) |  |  |  |  |
| PMS2 | 22 (75.86%) | 20 (80.00%) | 14 (87.50%) | 7 (77.78%) | 7 (100.00%) | 11 (100.00%) | 7 (87.50%) | 4 (100.00%) | 15 (68.18%) | 9 (64.29%) | 7 (87.50%) | 3 (60.00%) |  |  |  |  |
| PD-L1 CPS (n=34, 27, 5, 2) | n=6 | n=14 | n=7 | n=7 | n=5 | n=10 | n=6 | n=6 | n=1 | n=3 | n=1 | n=0 | n=0 | n=1 | n=0 | n=1 |
| Negative | 4 (66.67%) | 4 (28.57%) | 2 (28.57%) | 3 (42.86%) | 3 (60.00%) | 3 (30.00%) | 1 (16.67%) | 2 (33.33%) | 1 (100.00%) | 1 (33.33%) | 1 (100.00%) | / | / | 0 (0.00%) | / | 1 (100.00%) |
| Positive | 2 (33.33%) | 10 (71.43%) | 5 (71.43%) | 4 (57.14%) | 2 (40.00%) | 7 (70.00%) | 5 (83.33%) | 4 (66.67%) | 0 (0%) | 2 (66.67%) | 0 (0.00%) | / | / | 1 (100.00%) | / | 0 (0.00%) |
| HER-2 (n=2220, 802, 1371, 47) | n=592 | n=561 | n=217 | n=319 | n=166 | n=281 | n=112 | n=185 | n=425 | n=265 | n=95 | n=116 | n=1 | n=15 | n=10 | n=18 |
| Negative | 580 (97.97%) | 543 (96.79%) | 207 (95.39%) | 299 (93.73%) | 158 (95.18%) | 268 (95.37%) | 103 (91.96%) | 171 (92.43%) | 421 (99.06%) | 260 (98.11%) | 94 (98.95%) | 110 (94.83%) |  | 15 (100.00%) | 10 (100.00%) | 18 (100.00%) |
| Positive | 12 (2.03%) | 18 (3.21%) | 10 (4.61%) | 20 (6.27%) | 8 (4.82%) | 13 (4.63%) | 9 (8.04%) | 14 (7.57%) | 4 (0.94%) | 5 (1.89%) | 1 (1.05%) | 6 (5.17%) |  | 0 (0.00%) | 0 (0.00%) | 0 (0.00%) |
| KI-67 (n=2357, 799, 1377, 181) | n=601 | n=602 | n=231 | n=386 | n=165 | n=282 | n=113 | n=182 | n=432 | n=264 | n=94 | n=114 | n=4 | n=56 | n=24 | n=90 |
| <5% | 5 (0.83%) | 9 (1.50%) | 3 (1.3%) | 4 (1.04%) | 1 (0.61%) | 3 (1.06%) | 1 (0.88%) | 1 (0.55%) | 3 (0.69%) | 3 (1.14%) | 0 (0.00%) | 0 (0%) | 1 (25.00%) | 3 (5.36%) | 2 (8.33%) | 3 (3.33%) |
| 5-25% | 10 (1.66%) | 32 (5.32%) | 4 (1.73%) | 33 (8.55%) | 7 (4.24%) | 7 (2.48%) | 2 (1.77%) | 6 (3.3%) | 3 (0.69%) | 2 (0.76%) | 0 (0.00%) | 2 (1.75%) | 0 (0.00%) | 23 (41.07%) | 2 (8.33%) | 25 (27.78%) |
| >25% | 586 (97.50%) | 561 (93.19%) | 224 (96.97%) | 349 (90.41%) | 157 (95.15%) | 272 (96.45%) | 110 (97.35%) | 175 (96.15%) | 426 (98.61%) | 259 (98.11%) | 94 (100.00%) | 112 (98.25%) | 3 (75.00%) | 30 (53.57%) | 20 (83.33%) | 62 (68.89%) |
| KNB Mutations (n=853, 13, 836, 4) |  |  |  |  |  |  |  |  |  |  |  |  |  |  |  |  |
| KRAS | n=262 | n=158 | n=64 | n=78 | n=3 | n=3 | n=3 | n=4 | n=259 | n=154 | n=61 | n=72 | n=0 | n=1 | n=0 | n=2 |
| Mutation | 132 (50.38%) | 81 (51.27%) | 38 (59.38%) | 41 (52.56%) | 0 (0.00%) | 0 (0.00%) | 0 (0.00%) | 1 (25.00%) | 132 (50.97%) | 81 (52.60%) | 38 (62.30%) | 38 (52.78%) | / | 0 (0.00%) | / | 2 (100.00%) |
| Negative | 130 (49.62%) | 77 (48.73%) | 26 (40.63%) | 37 (47.44%) | 3 (100.00%) | 3 (100.00%) | 3 (100.00%) | 3 (75.00%) | 127 (49.03%) | 73 (47.40%) | 23 (37.70%) | 34 (47.22%) | / | 1 (100.00%) | / | 0 (0.00%) |
| NRAS |  |  |  |  |  |  |  |  |  |  |  |  |  |  |  |  |
| Mutation | 11 (4.20%) | 4 (2.53%) | 1 (1.56%) | 2 (2.56%) | 0 (0.00%) | 0 (0.00%) | 0 (0.00%) | 0 (0.00%) | 11 (4.25%) | 4 (2.60%) | 1 (1.64%) | 2 (2.78%) | / | 0 (0.00%) | / | 0 (0.00%) |
| Negative | 251 (95.80%) | 154 (97.47%) | 63 (98.44%) | 76 (97.44%) | 3 (100.00%) | 3 (100.00%) | 3 (100.00%) | 4 (100.00%) | 248 (95.75%) | 150 (97.40%) | 60 (98.36%) | 70 (97.22%) | / | 1 (100.00%) | / | 2 (100.00%) |
| BRAF |  |  |  |  |  |  |  |  |  |  |  |  |  |  |  |  |
| Mutation | 5 (1.91%) | 9 (5.70%) | 3 (4.69%) | 5 (6.41%) | 1 (33.33%) | 0 (0.00%) | 0 (0.00%) | 1 (25.00%) | 4 (1.54%) | 9 (5.84%) | 3 (4.92%) | 4 (5.56%) | / | 0 (0.00%) | / | 0 (0.00%) |
| Negative | 257 (98.09%) | 149 (94.30%) | 61 (95.31%) | 73 (93.59%) | 2 (66.67%) | 3 (100.00%) | 3 (100.00%) | 3 (75.00%) | 255 (98.46%) | 145 (94.16%) | 58 (95.08%) | 68 (94.44%) | / | 1 (100.00%) | / | 2 (100.00%) |

**Supplemental Table S1C.** Expression of Trop2 among different clinicopathological features: classification based on Trop2 intensity.

| **Characteristics** | **All cases (n=2370)** | | | **Gastric cancer (n=804)** | | | **Colorectal cancer (n=1384)** | | | **Pancreatic cancer (n=182)** | | |
| --- | --- | --- | --- | --- | --- | --- | --- | --- | --- | --- | --- | --- |
|  | **Weak (%)** | **Medium (%)** | **Strong (%)** | **Weak (%)** | **Medium (%)** | **Strong (%)** | **Weak (%)** | **Medium (%)** | **Strong (%)** | **Weak (%)** | **Medium (%)** | **Strong (%)** |
| Gender | n=1198 | n=186 | n=446 | n=488 | n=72 | n=186 | n=610 | n=103 | n=196 | n=100 | n=11 | n=64 |
| Male | 774 (64.61%) | 114 (61.29%) | 285 (63.90%) | 346 (70.90%) | 46 (63.89%) | 140 (75.27%) | 360 (59.02%) | 62 (60.19%) | 112 (57.14%) | 68 (68.00%) | 6 (54.55%) | 33 (51.56%) |
| Female | 424 (35.39%) | 72 (38.71%) | 161 (36.10%) | 142 (29.10%) | 26 (36.11%) | 46 (24.73%) | 250 (40.98%) | 41 (39.81%) | 84 (42.86%) | 32 (32.00%) | 5 (45.45%) | 31 (48.44%) |
| Age (years) |  |  |  |  |  |  |  |  |  |  |  |  |
| <65 | 688 (57.43%) | 116 (62.37%) | 251 (56.28%) | 274 (56.15%) | 47 (65.28%) | 87 (46.77%) | 356 (58.36%) | 60 (58.25%) | 122 (62.24%) | 58 (58.00%) | 9 (81.82%) | 42 (65.63%) |
| ≥65 | 510 (42.57%) | 70 (37.63%) | 195 (43.72%) | 214 (43.85%) | 25 (34.72%) | 99 (53.23%) | 254 (41.64%) | 43 (41.75%) | 74 (37.76%) | 42 (42.00%) | 2 (18.18%) | 22 (34.38%) |
| Tumor sites |  |  |  |  |  |  |  |  |  |  |  |  |
| Stomach | 488 (40.73%) | 72 (38.71%) | 186 (41.70%) | 488 (100.00%) | 72 (100.00%) | 186 (100.00%) |  |  |  |  |  |  |
| Gastroesophageal junction |  |  |  | 13 (2.66%) | 3 (4.17%) | 2 (1.08%) |  |  |  |  |  |  |
| Cardia |  |  |  | 89 (18.24%) | 14 (19.44%) | 37 (19.89%) |  |  |  |  |  |  |
| Gastric body |  |  |  | 114 (23.36%) | 17 (23.61%) | 43 (23.12%) |  |  |  |  |  |  |
| Gastric fundus |  |  |  | 15 (3.07%) | 2 (2.78%) | 6 (3.23%) |  |  |  |  |  |  |
| Gastric antrum |  |  |  | 182 (37.30%) | 22 (30.56%) | 63 (33.87%) |  |  |  |  |  |  |
| Lesser curvature |  |  |  | 61 (12.50%) | 9 (12.50%) | 29 (15.59%) |  |  |  |  |  |  |
| Pylorus |  |  |  | 3 (0.61%) | 2 (2.78%) | 1 (0.54%) |  |  |  |  |  |  |
| Gastric remnant |  |  |  | 11 (2.25%) | 3 (4.17%) | 5 (2.69%) |  |  |  |  |  |  |
| Colorectum | 610 (50.92%) | 103 (55.38%) | 196 (43.95%) |  |  |  | 610 (100.00%) | 103 (100.00%) | 196 (100.00%) |  |  |  |
| Colon |  |  |  |  |  |  | 220 (36.07%) | 35 (33.98%) | 64 (32.65%) |  |  |  |
| Sigmoid colon |  |  |  |  |  |  | 95 (15.57%) | 14 (13.59%) | 28 (14.29%) |  |  |  |
| Rectum |  |  |  |  |  |  | 293 (48.03%) | 52 (50.49%) | 101 (51.53%) |  |  |  |
| Cecum |  |  |  |  |  |  | 2 (0.33%) | 2 (1.94%) | 3 (1.53%) |  |  |  |
| Pancreas | 100 (8.35%) | 11 (5.91%) | 64 (14.35%) |  |  |  |  |  |  | 100 (100.00%) | 11 (100.00%) | 64 (100.00%) |
| Histological classification |  |  |  |  |  |  |  |  |  |  |  |  |
| Adenocarcinoma | 1185 (98.91%) | 184 (98.92%) | 442 (99.10%) | 480 (98.36%) | 70 (97.22%) | 182 (97.85%) | 608 (99.67%) | 103 (100.00%) | 196 (100.00%) | 97 (97.00%) | 11 (100.00%) | 64 (100.00%) |
| Others | 13 (1.09%) | 2 (1.08%) | 4 (0.90%) | 8 (1.64%) | 2 (2.78%) | 4 (2.15%) | 2 (0.33%) | 0 (0.00%) | 0 (0.00%) | 3 (3.00%) | 0 (0.00%) | 0 (0.00%) |
| Signet ring cell carcinoma components (SRCC) |  |  |  |  |  |  |  |  |  |  |  |  |
| Yes | 97 (8.10%) | 26 (13.98%) | 45 (10.09%) | 85 (17.42%) | 22 (30.56%) | 41 (22.04%) | 11 (1.80%) | 4 (3.88%) | 3 (1.53%) | 1 (1.00%) | 0 (0.00%) | 1 (1.56%) |
| No | 1101 (91.90%) | 160 (86.02%) | 401 (89.91%) | 403 (82.58%) | 50 (69.44%) | 145 (77.96%) | 599 (98.20%) | 99 (96.12%) | 193 (98.47%) | 99 (99.00%) | 11 (100.00%) | 63 (98.44%) |
| Mucinous adenocarcinoma components (MAC) |  |  |  |  |  |  |  |  |  |  |  |  |
| Yes | 101 (8.43%) | 19 (10.22%) | 45 (10.09%) | 18 (3.69%) | 4 (5.56%) | 11 (5.91%) | 80 (13.11%) | 15 (14.56%) | 31 (15.82%) | 3 (3.00%) | 0 (0.00%) | 3 (4.69%) |
| No | 1097 (91.57%) | 167 (89.78%) | 401 (89.91%) | 470 (96.31%) | 68 (94.44%) | 175 (94.09%) | 530 (86.89%) | 88 (85.44%) | 165 (84.18%) | 97 (97.00%) | 11 (100.00%) | 61 (95.31%) |
| Lauren classification (n=671, 671, 0, 0) | n=410 | n=59 | n=154 | n=410 | n=59 | n=154 | / | / | / | / | / | / |
| Intestinal type | 95 (23.17%) | 16 (27.12%) | 46 (29.87%) | 95 (23.17%) | 16 (27.12%) | 46 (29.87%) |  |  |  |  |  |  |
| Diffuse type | 108 (26.34%) | 18 (30.51%) | 25 (16.23%) | 108 (26.34%) | 18 (30.51%) | 25 (16.23%) |  |  |  |  |  |  |
| Mixed type | 184 (44.88%) | 23 (38.98%) | 77 (50.00%) | 184 (44.88%) | 23 (38.98%) | 77 (50.00%) |  |  |  |  |  |  |
| Indeterminate type | 23 (5.61%) | 2 (3.39%) | 6 (3.90%) | 23 (5.61%) | 2 (3.39%) | 6 (3.90%) |  |  |  |  |  |  |
| Differentiation degree |  |  |  |  |  |  |  |  |  |  |  |  |
| Poor | 703 (58.68%) | 117 (62.90%) | 273 (61.21%) | 393 (80.53%) | 60 (83.33%) | 142 (76.34%) | 249 (40.82%) | 50 (48.54%) | 88 (44.90%) | 61 (61.00%) | 7 (63.64%) | 43 (67.19%) |
| Moderate | 389 (32.47%) | 61 (32.80%) | 132 (29.60%) | 48 (9.84%) | 10 (13.89%) | 23 (12.37%) | 313 (51.31%) | 48 (46.60%) | 96 (48.98%) | 28 (28.00%) | 3 (27.27%) | 13 (20.31%) |
| Well | 106 (8.85%) | 8 (4.30%) | 41 (9.19%) | 47 (9.63%) | 2 (2.78%) | 21 (11.29%) | 48 (7.87%) | 5 (4.85%) | 12 (6.12%) | 11 (11.00%) | 1 (9.09%) | 8 (12.50%) |
| T stage |  |  |  |  |  |  |  |  |  |  |  |  |
| 1 | 169 (14.11%) | 21 (11.29%) | 55 (12.33%) | 126 (25.82%) | 12 (16.67%) | 34 (18.28%) | 31 (5.08%) | 6 (5.83%) | 9 (4.59%) | 12 (12.00%) | 3 (27.27%) | 12 (18.75%) |
| 2 | 170 (14.19%) | 29 (15.59%) | 77 (17.26%) | 48 (9.84%) | 11 (15.28%) | 23 (12.37%) | 68 (11.15%) | 12 (11.65%) | 21 (10.71%) | 54 (54.00%) | 6 (54.55%) | 33 (51.56%) |
| 3 | 552 (46.08%) | 97 (52.15%) | 175 (39.24%) | 161 (32.99%) | 29 (40.28%) | 60 (32.26%) | 376 (61.64%) | 67 (65.05%) | 104 (53.06%) | 15 (15.00%) | 1 (9.09%) | 11 (17.19%) |
| 4 | 217 (18.11%) | 25 (13.44%) | 88 (19.73%) | 117 (23.98%) | 13 (18.06%) | 50 (26.88%) | 87 (14.26%) | 12 (11.65%) | 35 (17.86%) | 13 (13.00%) | 0 (0.00%) | 3 (4.69%) |
| NA | 90 (7.51%) | 14 (7.53%) | 51 (11.43%) | 36 (7.38%) | 7 (9.72%) | 19 (10.22%) | 48 (7.87%) | 6 (5.83%) | 27 (13.78%) | 6 (6.00%) | 1 (9.09%) | 5 (7.81%) |
| N stage |  |  |  |  |  |  |  |  |  |  |  |  |
| 0 | 515 (42.99%) | 81 (43.55%) | 184 (41.26%) | 202 (41.39%) | 31 (43.06%) | 75 (40.32%) | 256 (41.97%) | 46 (44.66%) | 79 (40.31%) | 57 (57.00%) | 4 (36.36%) | 30 (46.88%) |
| 1 | 274 (22.87%) | 47 (25.27%) | 99 (22.20%) | 74 (15.16%) | 9 (12.50%) | 27 (14.52%) | 170 (27.87%) | 32 (31.07%) | 50 (25.51%) | 30 (30.00%) | 6 (54.55%) | 22 (34.38%) |
| 2 | 211 (17.61%) | 28 (15.05%) | 84 (18.83%) | 68 (13.93%) | 9 (12.50%) | 37 (19.89%) | 136 (22.30%) | 19 (18.45%) | 40 (20.41%) | 7 (7.00%) | 0 (0.00%) | 7 (10.94%) |
| 3 | 108 (9.02%) | 16 (8.60%) | 28 (6.28%) | 108 (22.13%) | 16 (22.22%) | 28 (15.05%) | / | / | / | / | / | / |
| NA | 90 (7.51%) | 14 (7.53%) | 51 (11.43%) | 36 (7.38%) | 7 (9.72%) | 19 (10.22%) | 48 (7.87%) | 6 (5.83%) | 27 (13.78%) | 6 (6.00%) | 1 (9.09%) | 5 (7.81%) |
| M stage |  |  |  |  |  |  |  |  |  |  |  |  |
| 0 | 1006 (83.97%) | 158 (84.95%) | 359 (80.49%) | 426 (87.30%) | 62 (86.11%) | 157 (84.41%) | 502 (82.30%) | 89 (86.41%) | 155 (79.08%) | 78 (78.00%) | 7 (63.64%) | 47 (73.44%) |
| 1 | 150 (12.52%) | 23 (12.37%) | 72 (16.14%) | 42 (8.61%) | 8 (11.11%) | 24 (12.90%) | 88 (14.43%) | 11 (10.68%) | 33 (16.84%) | 20 (20.00%) | 4 (36.36%) | 15 (23.44%) |
| NA | 42 (3.51%) | 5 (2.69%) | 15 (3.36%) | 20 (4.10%) | 2 (2.78%) | 5 (2.69%) | 20 (3.28%) | 3 (2.91%) | 8 (4.08%) | 2 (2.00%) | 0 (0.00%) | 2 (3.13%) |
| Stage |  |  |  |  |  |  |  |  |  |  |  |  |
| I | 261 (21.79%) | 35 (18.82%) | 86 (19.28%) | 147 (30.12%) | 20 (27.78%) | 41 (22.04%) | 76 (12.46%) | 12 (11.65%) | 22 (11.22%) | 38 (38.00%) | 3 (27.27%) | 23 (35.94%) |
| II | 279 (23.29%) | 57 (30.65%) | 120 (26.91%) | 89 (18.24%) | 19 (26.39%) | 47 (25.27%) | 168 (27.54%) | 34 (33.01%) | 56 (28.57%) | 22 (22.00%) | 4 (36.36%) | 17 (26.56%) |
| III | 466 (38.90%) | 66 (35.48%) | 153 (34.30%) | 190 (38.93%) | 23 (31.94%) | 69 (37.10%) | 258 (42.30%) | 43 (41.75%) | 77 (39.29%) | 18 (18.00%) | 0 (0.00%) | 7 (10.94%) |
| IV | 150 (12.52%) | 23 (12.37%) | 72 (16.14%) | 42 (8.61%) | 8 (11.11%) | 24 (12.90%) | 88 (14.43%) | 11 (10.68%) | 33 (16.84%) | 20 (20.00%) | 4 (36.36%) | 15 (23.44%) |
| NA | 42 (3.51%) | 5 (2.69%) | 15 (3.36%) | 20 (4.10%) | 2 (2.78%) | 5 (2.69%) | 20 (3.28%) | 3 (2.91%) | 8 (4.08%) | 2 (2.00%) | 0 (0.00%) | 2 (3.13%) |
| Metastasis |  |  |  |  |  |  |  |  |  |  |  |  |
| Yes | 150 (12.52%) | 23 (12.37%) | 72 (16.14%) | 42 (8.61%) | 8 (11.11%) | 24 (12.90%) | 88 (14.43%) | 11 (10.68%) | 33 (16.84%) | 20 (20.00%) | 4 (36.36%) | 15 (23.44%) |
| No | 1048 (87.48%) | 163 (87.63%) | 374 (83.86%) | 446 (91.39%) | 64 (88.89%) | 162 (87.10%) | 522 (85.57%) | 92 (89.32%) | 163 (83.16%) | 80 (80.00%) | 7 (63.64%) | 49 (76.56%) |
| Vascular invasion (VI, n=2109, 723, 1256, 130) | n=1069 | n=169 | n=379 | n=422 | n=65 | n=163 | n=556 | n=98 | n=168 | n=71 | n=6 | n=48 |
| Yes | 392 (36.67%) | 75 (44.38%) | 189 (49.87%) | 197 (44.57%) | 33 (50.77%) | 93 (57.06%) | 177 (31.83%) | 42 (42.86%) | 76 (45.24%) | 18 (25.35%) | 0 (0.00%) | 20 (41.67%) |
| No | 677 (63.33%) | 94 (55.62%) | 190 (50.13%) | 245 (55.43%) | 32 (49.23%) | 70 (42.94%) | 379 (68.17%) | 56 (57.14%) | 92 (54.76%) | 53 (74.65%) | 6 (100.00%) | 28 (58.33%) |
| Perineural invasion (PNI, n=2109, 723, 1256, 130) | n=1069 | n=169 | n=379 | n=422 | n=65 | n=163 | n=556 | n=98 | n=168 | n=71 | n=6 | n=48 |
| Yes | 551 (51.54%) | 92 (54.44%) | 235 (62.01%) | 223 (50.45%) | 37 (56.92%) | 101 (61.96%) | 266 (47.84%) | 50 (51.02%) | 90 (53.57%) | 62 (87.32%) | 5 (83.33%) | 44 (91.67%) |
| No | 518 (48.46%) | 77 (45.56%) | 144 (37.99%) | 219 (49.55%) | 28 (43.08%) | 62 (38.04%) | 290 (52.16%) | 48 (48.98%) | 78 (46.43%) | 9 (12.68%) | 1 (16.67%) | 4 (8.33%) |
| MMR status |  |  |  |  |  |  |  |  |  |  |  |  |
| pMMR | 1142 (95.33%) | 179 (96.24%) | 430 (96.41%) | 467 (95.70%) | 71 (98.61%) | 178 (95.70%) | 575 (94.26%) | 97 (94.17%) | 188 (95.92%) | 100 (100.00%) | 11 (100.00%) | 64 (100.00%) |
| dMMR | 56 (4.67%) | 7 (3.76%) | 16 (3.59%) | 21 (4.30%) | 1 (1.39%) | 8 (4.30%) | 35 (5.74%) | 6 (5.83%) | 8 (4.08%) | 0 (0.00%) | 0 (0.00%) | 0 (0.00%) |
| Functional defects of tumor mismatch repair genes (n=123, 39, 83, 1) | n=56 | n=7 | n=16 | n=21 | n=1 | n=8 | n=35 | n=6 | n=8 | / | / | / |
| MSH2 | 7 (12.50%) | 3 (42.86%) | 1 (6.25%) | 0 (0.00%) | 0 (0.00%) | 0 (0.00%) | 7 (20.00%) | 3 (50.00%) | 1 (12.50%) |  |  |  |
| MLH1 | 40 (71.43%) | 3 (42.86%) | 15 (93.75%) | 19 (90.48%) | 1 (100.00%) | 8 (100.00%) | 21 (60.00%) | 2 (33.33%) | 7 (87.50%) |  |  |  |
| MSH6 | 10 (17.86%) | 2 (28.57%) | 1 (6.25%) | 0 (0.00%) | 0 (0.00%) | 0 (0.00%) | 10 (28.57%) | 2 (33.33%) | 1 (12.50%) |  |  |  |
| PMS2 | 44 (78.57%) | 4 (57.14%) | 15 (93.75%) | 20 (95.24%) | 1 (100.00%) | 8 (100.00%) | 24 (68.57%) | 3 (50.00%) | 7 (87.50%) |  |  |  |
| PD-L1 CPS (n=34, 27, 5, 2) | n=20 | n=2 | n=12 | n=16 | n=1 | n=10 | n=2 | n=1 | n=2 | n=2 | n=0 | n=0 |
| Negative | 7 (35.00%) | 0 (0.00%) | 6 (50.00%) | 5 (31.25%) | 0 (0.00%) | 4 (40.00%) | 1 (50.00%) | 0 (0.00%) | 2 (100.00%) | 1 (50.00%) | / | / |
| Positive | 13 (65.00%) | 2 (100%) | 6 (50.00%) | 11 (68.75%) | 1 (100.00%) | 6 (60.00%) | 1 (50.00%) | 1 (100.00%) | 0 (0.00%) | 1 (50.00%) | / | / |
| HER-2 (n=2220, 802, 1371, 47) | n=1115 | n=179 | n=395 | n=487 | n=72 | n=185 | n=603 | n=103 | n=195 | n=25 | n=4 | n=15 |
| Negative | 1078 (96.68%) | 171 (95.53%) | 380 (96.20%) | 459 (94.25%) | 67 (93.06%) | 174 (94.05%) | 594 (98.51%) | 100 (97.09%) | 191 (97.95%) | 25 (100.00%) | 4 (100.00%) | 15 (100.00%) |
| Positive | 37 (3.32%) | 8 (4.47%) | 15 (3.80%) | 28 (5.75%) | 5 (6.94%) | 11 (5.95%) | 9 (1.49%) | 3 (2.91%) | 4 (2.05%) | 0 (0.00%) | 0 (0.00%) | 0 (0.00%) |
| KI-67 (n=2357, 799, 1377, 181) | n=1194 | n=185 | n=441 | n=486 | n=71 | n=185 | n=608 | n=103 | n=193 | n=100 | n=11 | n=63 |
| <5% | 14 (1.17%) | 2 (1.08%) | 5 (1.13%) | 5 (1.03%) | 0 (0.00%) | 1 (0.54%) | 3 (0.49%) | 2 (1.94%) | 1 (0.52%) | 6 (6.00%) | 0 (0.00%) | 3 (4.76%) |
| 5-25% | 55 (4.61%) | 7 (3.78%) | 17 (3.85%) | 17 (3.50%) | 3 (4.23%) | 2 (1.08%) | 6 (0.99%) | 0 (0.00%) | 1 (0.52%) | 32 (32.00%) | 4 (36.36%) | 14 (22.22%) |
| >25% | 1125 (94.22%) | 176 (95.14%) | 419 (95.01%) | 464 (95.47%) | 68 (95.77%) | 182 (98.38%) | 599 (98.52%) | 101 (98.06%) | 191 (98.96%) | 62 (62.00%) | 7 (63.64%) | 46 (73.02%) |
| KNB Mutations (n=853, 13, 836, 4) |  |  |  |  |  |  |  |  |  |  |  |  |
| KRAS | n=373 | n=68 | n=121 | n=7 | n=1 | n=5 | n=365 | n=67 | n=114 | n=1 | n=0 | n=2 |
| Mutation | 194 (52.01%) | 35 (51.47%) | 63 (52.07%) | 0 (0.00%) | 0 (0.00%) | 1 (20.00%) | 194 (53.15%) | 35 (52.24%) | 60 (52.63%) | 0 (0.00%) | / | 2 (100.00%) |
| Negative | 179 (47.99%) | 33 (48.53%) | 58 (47.93%) | 7 (100.00%) | 1 (100.00%) | 4 (80.00%) | 171 (46.85%) | 32 (47.76%) | 54 (47.37%) | 1 (100.00%) | / | 0 (0.00%) |
| NRAS |  |  |  |  |  |  |  |  |  |  |  |  |
| Mutation | 14 (3.75%) | 1 (1.47%) | 3 (2.48%) | 0 (0.00%) | 0 (0.00%) | 0 (0.00%) | 14 (3.84%) | 1 (1.49%) | 3 (2.63%) | 0 (0.00%) | / | 0 (0.00%) |
| Negative | 359 (96.25%) | 67 (98.53%) | 118 (97.52%) | 7 (100.00%) | 1 (100.00%) | 5 (100.00%) | 351 (96.16%) | 66 (98.51%) | 111 (97.37%) | 1 (100.00%) | / | 2 (100.00%) |
| BRAF |  |  |  |  |  |  |  |  |  |  |  |  |
| Mutation | 15 (4.02%) | 3 (4.41%) | 4 (3.31%) | 1 (14.29%) | 0 (0.00%) | 1 (20.00%) | 14 (3.84%) | 3 (4.48%) | 3 (2.63%) | 0 (0.00%) | / | 0 (0.00%) |
| Negative | 358 (95.98%) | 65 (95.59%) | 117 (96.69%) | 6 (85.71%) | 1 (100.00%) | 4 (80.00%) | 351 (96.16%) | 64 (95.52%) | 111 (97.37%) | 1 (100.00%) | / | 2 (100.00%) |
